# Pro-Apoptotic and Anti-Invasive Properties Underscore the Tumor-Suppressing Impact of Myoglobin on a Subset of Human Breast Cancer Cells

**DOI:** 10.3390/ijms231911483

**Published:** 2022-09-29

**Authors:** Mostafa A. Aboouf, Julia Armbruster, Markus Thiersch, Franco Guscetti, Glen Kristiansen, Peter Schraml, Anne Bicker, Ruben Petry, Thomas Hankeln, Max Gassmann, Thomas A. Gorr

**Affiliations:** 1 Institute of Veterinary Physiology, Vetsuisse Faculty, University of Zurich, 8057 Zurich, Switzerland; 2 Center for Clinical Studies, Vetsuisse Faculty, University of Zurich, 8057 Zurich, Switzerland; 3 Department of Biochemistry, Faculty of Pharmacy, Ain Shams University, Cairo 11566, Egypt; 4 Zurich Center for Integrative Human Physiology (ZIHP), University of Zurich, 8057 Zurich, Switzerland; 5 Institute of Veterinary Pathology, Vetsuisse Faculty, University of Zurich, 8057 Zurich, Switzerland; 6 Institute of Pathology, University Hospital Bonn, University of Bonn, D-53127 Bonn, Germany; 7 Department of Pathology and Molecular Pathology, University Hospital Zurich, University of Zurich, 8091 Zurich, Switzerland; 8 Institute of Organismic and Molecular Evolution, Molecular Genetics and Genome Analysis, Johannes Gutenberg University, D-55099 Mainz, Germany; 9 University Medical Center of the Johannes Gutenberg University Mainz, I. Medical Clinic, Langenbeckstrasse 1, D-55131 Mainz, Germany

**Keywords:** tumorigenesis, migration, apoptosis, ROS, estrogen receptor, hypoxia, chemotherapy, radiation, p53

## Abstract

The expression of myoglobin (MB), well known as the oxygen storage and transport protein of myocytes, is a novel hallmark of the luminal subtype in breast cancer patients and correlates with better prognosis. The mechanisms by which MB impacts mammary tumorigenesis are hitherto unclear. We aimed to unravel this role by using CRISPR/Cas9 technology to generate MB-deficient clones of MCF7 and SKBR3 breast cancer cell lines and subsequently characterize them by transcriptomics plus molecular and functional analyses. As main findings, loss of MB at normoxia upregulated the expression of cell cyclins and increased cell survival, while it prevented apoptosis in MCF7 cells. Additionally, MB-deficient cells were less sensitive to doxorubicin but not ionizing radiation. Under hypoxia, the loss of MB enhanced the partial epithelial to mesenchymal transition, thus, augmenting the migratory and invasive behavior of cells. Notably, in human invasive mammary ductal carcinoma tissues, MB and apoptotic marker levels were positively correlated. In addition, MB protein expression in invasive ductal carcinomas was associated with a positive prognostic value, independent of the known tumor suppressor p53. In conclusion, we provide multiple lines of evidence that endogenous MB in cancer cells by itself exerts novel tumor-suppressive roles through which it can reduce cancer malignancy.

## 1. Introduction

Myoglobin (MB) is well known as the heme-binding globin of the heart and skeletal muscles where it is present in the µM (terrestrial mammals) to mM (apnoe diving mammals) concentration range. There, MB functions as a temporary store of O_2_ and buffers short phases of the exercise-induced increase in aerobic metabolism by supplying O_2_ to the myocytes’ mitochondria [1,2,3]. MB was also reported to detoxify harmful reactive oxygen species (ROS) [4] as well as to maintain nitric oxide (NO^•^) homeostasis in cardiomyocytes by either scavenging or producing the radical in normoxic or hypoxic environments, respectively [5,6]. Both ROS and NO^•^ are important cell signaling mediators, implying that MB broadly regulates mammalian cell behavior via various cell signaling pathways. Recently, MB was discovered to also be expressed in different tumor types, including multiple epithelial cancers (e.g., breast cancer, prostate cancer, non-small cell lung cancer (NSCLC), etc.) as well as in the leukemic bone marrow [7,8,9,10,11,12]. Being expressed at sub-µM levels in these tumor types, however, it is still unclear whether this low-level abundance is sufficient for the effective oxygenation of cells [13,14]. While the prominent occurrence of MB in muscle cells is known to prevent apoptosis and to help detoxify chemotherapeutics such as doxorubicin to non-lethal metabolites [15], these features are still to be demonstrated for malignant tissues. At concentrations that lie perhaps 1000 times below those of myocytes, MB in cancer cells might indeed harbor a set of novel and rather catalytic functions.

A strong hint to a new set of MB functions might be deduced from its ectopic expression site. In the healthy breast parenchyma of human subjects and the sub-cohort of MB-positive breast and prostate human cancer [8,9,10], MB was expressed not in the contractile myoepithelial layer but exclusively in the inner secretory luminal epithelium, whose malignant transformation marks the origin of the vast majority of primary human breast carcinomas [16]. Increasing evidence strengthens the notion that oxygenated MB (oxymyoglobin, MBO_2_) contributes to the production and secretion of lipid components into the milk by binding and transporting fatty acids [17,18], perhaps also in contexts of non-malignant luminal cells, i.e., those of mammary glands in healthy ‘mice and men’ [13]. Beyond the mammary gland, we recently demonstrated that the presence of MB in the brown adipose tissue (BAT) of mice appears to link oxygen and lipid-based thermogenic metabolism by shifting the lipid droplet (LD) equilibrium towards higher counts of smaller droplets (i.e., towards a browning phenotype) [19]. Importantly, MB in breast epithelia is, despite its low expression level, still of clinical relevance. In mammary carcinomas, MB was found to be expressed in around 40% of invasive ductal carcinomas where it positively correlates with a higher degree of tumor cell differentiation (luminal subtype), estrogen receptor positivity (ER+), and a significantly better prognostic outcome in ER+ or ER– breast cancer patients [8]. Hence, MB was validated as a novel luminal marker for breast cancer where it adds to the prognostic value of ER. In prostate cancer, MB was associated with androgen receptor expression, markers of tumor hypoxia, and a trend toward a prolonged recurrence-free survival [10]. In agreement with these observations, MB expression was detected in almost every second tumor from human patients with head and neck squamous cell carcinoma (HNSCC), again correlating with a favorable overall survival [9]. Conversely, overexpression of MB in NSCLC was associated with poor prognosis [11], suggesting a tumor-type specific role of MB in cancer cells.

At the molecular level, the expression of MB in human breast cancer (BrCa) cells can be controlled by mitogenic stimuli or oxidative stress [7]. Furthermore, we previously discovered three MB transcripts predominantly expressed in BrCa cells and breast tumors [20], which are under the control of a hypoxia-inducible enhancer/promoter [21] that can be silenced by hormonal treatment [8,20]. MB’s tumor suppressive biology was underscored by its transcriptional regulation in human BrCa cells [20,21,22]. In addition, in LNCaP prostate cancer cells, MB was directly or indirectly involved in the induction of apoptosis, TP21 expression, diminished migratory capacity, and possible induction of cell cycle arrest [22]. However, in MDA-MB 468 breast cancer cells, MB increased the migration [13,22], and its impact on apoptosis was inconclusive [22]. Whether these opposing observations can be attributed to differences in MB expression levels, tumor type or the existence of other functional mediators (such as hormonal receptors or p53 status) needs to be explored. In the current study, we, therefore, aimed to analyze MB’s role in governing various aspects of cellular vitality and therapeutic responsiveness specifically of breast cancer cells. We designed our experiments to decipher the role of MB in human BrCa cell lines MCF7 and SKBR3. We used CRISPR/Cas9 to generate MB wildtype (MB-WT) and MB knockout (MB-KO) cells, with which we analyzed the effect of the endogenous MB expression on apoptosis, cell proliferation, migration, and invasion, yet, in ways not directly related to the facilitated diffusion or storage of O_2_. The present study provides evidence along with mechanistic insights on the pivotal role played by endogenous MB in tumor suppression and opens the door to a potentially new anti-cancer intervention.

## 2. Results

**Figure 1:**
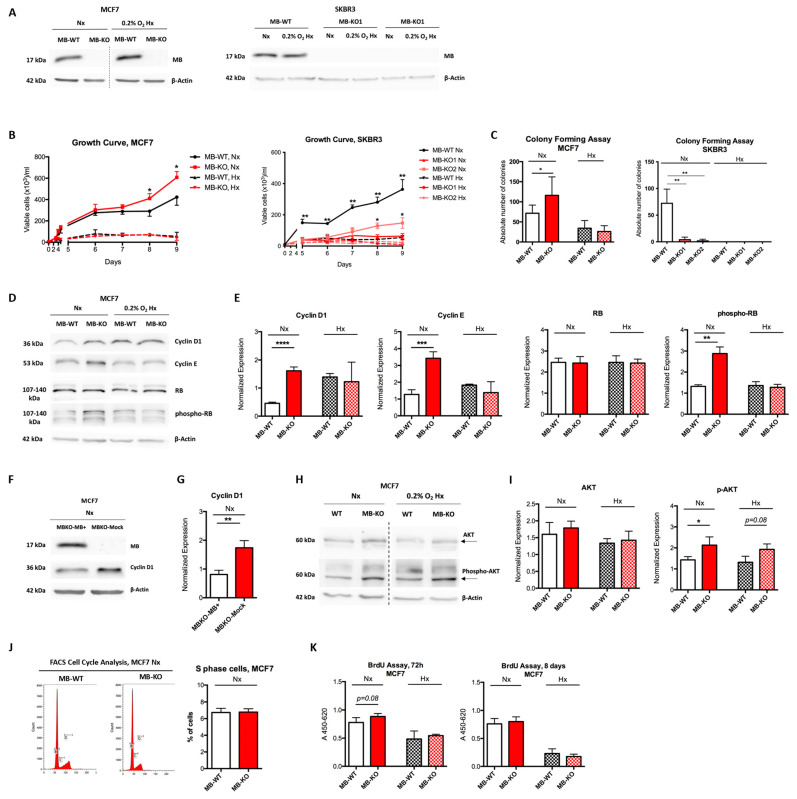
Myoglobin impacts breast cancer cell survival. (**A**) Representative Western blot stained for myoglobin (MB) (17 kDa) to verify the MB knockout (MB-KO) in MCF7 and SKBR3 cells. Cells were exposed to normoxia (Nx) or hypoxia (Hx, 0.2% O_2_) for 72 h prior to protein extraction (*n* = 4). (**B**) Growth curves of MB-KO vs. MB-WT of MCF7 (**left panel**) and SKBR3 (**right panel**) at Nx and 0.2% O_2_ Hx, respectively. Cells were stained with trypan blue, and dead cells defined by their blue appearance were excluded from counting (*n* = 3). (**C**) Colony assay performed using MB-WT and MB-KO clones of MCF7 (**left panel**) and SKBR3 (**right panel**). Cells were seeded at low confluency and incubated in Nx or 0.2% O_2_ Hx for 9 days. Colonies (>50 cells) stained with crystal violet were counted using a binocular (*n* = 5). (**D**) Representative Western blotting image of whole tissue lysate of MB-WT and MB-KO clones of MCF7 cells cultured at Nx or 0.2%O_2_ Hx for 72 h and stained for cyclin-D1 (33 kDa), cyclin E (53 kDa), retinoblastoma (RB) (130 kDa), phospho-retinoblastoma (p-RB) (130 kDa), and β-actin (42 kDa) used as the loading control (*n* = 4). (**E**) Band intensity of cyclin D1, cyclin E, RB, and phospho-RB proteins after Western blotting, from MB-WT (white) and MB-KO (red) MCF7 cells at Nx (empty bars) and 0.2%O_2_ Hx (dashed bars), was quantified using MCID Analysis 7.0 and normalized to β-actin (*n* = 4). (**F**) Representative Western blotting image of whole tissue lysate of MCF7 MB-KO cells transfected with MB+ or mCherry plasmids and stained for cyclin-D1 (33 kDa) and β-actin (42 kDa) used as the loading control (*n* = 3). (**G**) Band intensity of cyclin D1 after Western blotting, from MB-KO transfected with MB+ (white) or mCherry plasmids (red) cells at Nx was quantified using MCID Analysis 7.0 and normalized to β-actin (*n* = 3). (**H**) Representative Western blotting image (*n* = 3) of whole tissue lysate of MB-WT and MB-KO clones of MCF7 cells cultured at Nx or 0.2% O_2_ Hx for 72 h and stained for AKT (60 kDa), phospho-AKT (p-AKT) (60 kDa), and β-actin (42 kDa) used as the loading control. (**I**) Band intensity of AKT and phospho-AKT after Western blotting, from MB-WT (white) and MB-KO (red) cells at Nx (empty bars) and 0.2%O_2_ Hx (dashed bars), was quantified using MCID Analysis 7.0 and normalized to β-actin (*n* = 3). (**J**) FACS analysis of cell cycle status of MCF7 cells (B marks G1 phase cells, C marks S phase, and D marks G2/M phase cells) after culturing at Nx for 72 h and staining with Pyronin-Y and Hoechst stains and quantification of proportions of cells at S phase (*n* = 3). (**K**) BrdU incorporation assay of MB-WT (white) and MB-KO (red) clones of MCF7 cells cultured at Nx (empty bars) or 0.2% O_2_ Hx (dashed bars) for 72 h or 8 days. The absorbance of clones with anti-BrdU antibody for 4.5 h was measured at 450 nm and corrected at 620 nm (*n* = 4). Data are presented as the mean and standard error of mean and analyzed by Student’s *t*-test except for SKBR3 data in panel (**C**) by One-way ANOVA with Tukey’s multiple comparison post-hoc analysis * *p* < 0.05, ** *p* < 0.01, *** *p* < 0.001, **** *p* < 0.0001.

We generated MB-KO breast cancer cells of the MCF7 and SKBR3 human cell lines and compared their molecular and functional profiles to those of matching MB-WT control cells. The two BrCa cell lines were chosen for studying the tumor-specific effects of MB in a p53-WT/estrogen receptor (ER)-positive (MCF7) and a p53 gain-of-function (GoF)/ER-negative (SKBR3) background. As detailed in material and methods, all KO constructs were based on single cell clones to exploit cell models with an identical genetic background that only differ in *MB* with a specific knockout hit in each allele of the *MB* gene, which was verified on genomic DNA and the expected resultant truncated protein (Supplemental Figures S1 and S2). We detected no MB protein expression by Western blotting in the MB-KO clones (Figure 1A). In WT controls, MB protein was upregulated during prolonged (72 h) severe (0.2% O_2_) hypoxia in MCF7 but not in SKBR3 cells (Figure 1A).

### 2.1. Myoglobin Impacts Breast Cancer Cell Survival

Whether endogenous, rather than overexpressed, MB expression helps to regulate BrCa cell survival has never been investigated so far. To answer this, we analyzed the growth of cancer cell clones in normoxic (Nx, air atmosphere) and hypoxic (Hx, atmosphere with 0.2% O_2_) cultures using the Trypan blue viability exclusion assay. Nx, but not Hx, MB-KO MCF7 cells grew 33% faster than WT cells (Figure 1B), suggesting MBO_2_ either slows down cell proliferation or reduces the survival of cancer cells. In contrast, the loss of MB in SKBR3 cells lowered proliferation/survival under both normoxic and hypoxic conditions (Figure 1B). In addition, a clonogenic assay supported the previous results. Under Nx conditions, the loss of MB increased the number of colonies by 163% in MCF7 cells while lowering it by 680% in SKBR3 cells (Figure 1C). Due to the proliferative arrest of cells under severe hypoxia [23], far fewer colonies were formed at 0.2% O_2_ than under Nx conditions. We thus concluded that loss of MB increased MCF7 but decreased SKBR3 cell viability. and potentially, proliferation. To more specifically investigate the beneficial impact of MB basal expression on limiting cancer cell proliferation, we analyzed the expression of cyclin D1 and cyclin E in MCF7 cells, both regulating the cell division process in murine mammary tumors [24]. The loss of MB in Nx MCF7 cells increased the expression of cyclin D1 by 3.2 and cyclin E by 2.9-fold, respectively, (Figure 1D,E) and correlated with increased phosphorylation of retinoblastoma (RB) protein (Figure 1E). In contrast, the loss of MB in Hx MCF7 cells did not affect the expression of cyclin D1 or E (Figure 1D,E). Re-expressing MB in Nx KO cells resulted in diminished cyclin D1 expression (Figure 1F,G), thus, confirming MB-aided regulation of this specific cell cycle protein. As the expression of p27 and p21 proteins (Supplemental Figure S3A–C), as well as cyclin A or PCNA (Supplemental Figure S3D–F), was independent of MB expression, these data suggest MBO_2_ alters the G1/S phase transition independently from the p53 downstream proteins. Although levels of p-AKT were slightly increased in both normoxic and hypoxic MB-KO MCF7 cells (Figure 1H,I), loss of MB in normoxic MCF7 cells did not influence the percentage of S phase cells population (Figure 1J), suggesting that MB expression mainly regulates survival and not proliferation. Unchanged levels of uridine base uptake in normoxic MB-KO MCF7 cells cultured for either 3 or 8 days further supported that MB does not impact proliferation (Figure 1K). In conclusion, our data show that MB lessens the viability/survival, rather than reducing the proliferation, of oxygenated MCF7 cells.

### 2.2. Loss of Myoglobin Increases the Migratory Capacity of Hypoxic MCF7 Cancer Cells

**Figure 2:**
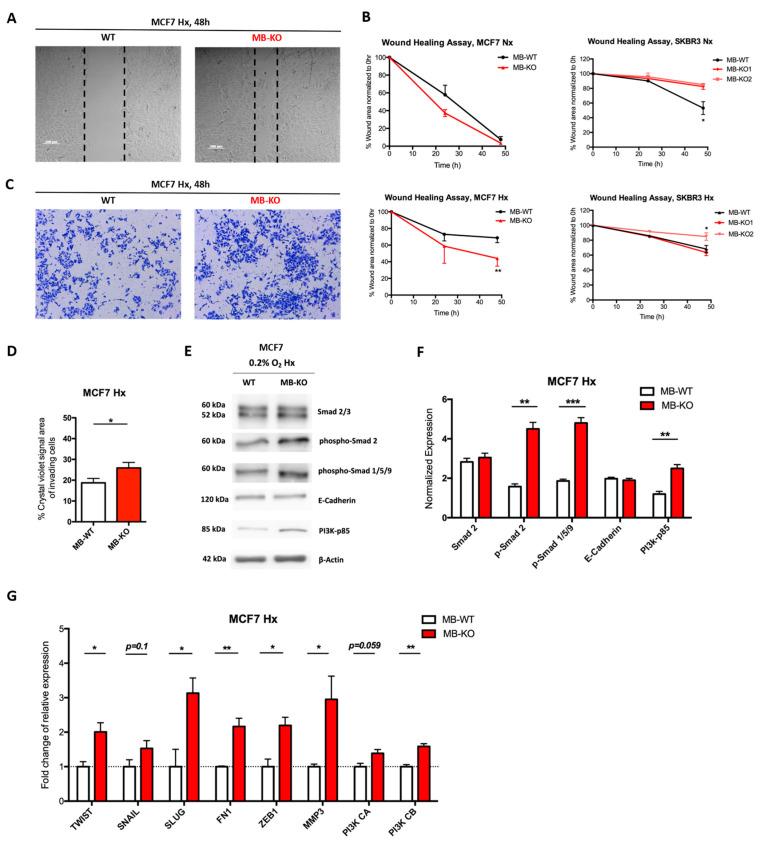
Loss of myoglobin increases the migratory capacity of hypoxic MCF7 cancer cells (**A**) Representative images of wound healing assay for MCF7 clones grown at 0.2% O_2_ hypoxia (Hx) in serum reduced medium for 48 h. A scratch was initiated into a monolayer of each cell line and cells were incubated in normoxia (Nx) or 0.2% O_2_ Hx. The % of the scratch area was measured and calculated after 24 and 48 h (*n* = 3). (**B**) Scatter plot of % scratch area (normalized to 0 h) against the time of incubation. MCF7 (left panels) and SKBR3 (right panels) wildtype (MB-WT, white) and MB knockout (MB-KO, red) clones (*n* = 3). (**C**) Representative images of Transwell invasion assay showing only invading wildtype and MB-KO MCF7 cells through a layer of Matrigel after culturing at 0.2% O_2_ Hx in Transwell for 48 h followed by fixation/staining with 0.6% glutaraldehyde and crystal violet solution. Magnification is 5x. (**D**) Quantitative representation of % of crystal violet signal area of invading cells of MB-WT (white) and MB-KO (red) of MCF7. Five random images from each well were captured and analyzed by ImageJ and the average of the signal area was calculated (*n* = 3). (**E**) Representative Western blotting image (*n* = 3) of whole tissue lysate of MB-WT and MB-KO clones of MCF7 cells cultured at 0.2% O_2_ Hx for 72 h and stained for Smad2/3 (60 and 52 kDa, respectively phospho-Smad2 (60 kDa phospho-Smad 1/5/9 (60 kDa), E-Cadherin (120 kDa), PI3K-p85 (85 kDa), and β-actin (42 kDa) used as the loading control. (**F**) Band intensity of Smad2, phospho-Smad2, phospho-Smad 1/5/9, E-Cadherin, and PI3K-p85 proteins after Western blotting, from MB-WT (white bars) and MB-KO (red bars) cells at 0.2% O_2_ Hx, was quantified using MCID Analysis 7.0 and normalized to β-actin (*n* = 3). (**G**) Relative mRNA expression levels of genes that regulate epithelial to mesenchymal transition: *TWIST, SNAIL, SLUG, FN1, ZEB1, MMP3, PI3K CA,* and *Pi3K CB*, quantified by qPCR and normalized to β-actin (*ACTB*) mRNA expression levels, from MB-WT (white) and MB-KO (red) clones of MCF7 cells cultured at 0.2% O_2_ Hx for 72 h. (*n* = 3 per group). Data are presented as mean and standard error of mean and analyzed by Student’s *t*-test * *p* < 0.05; ** *p* < 0.01, *** *p* < 0.001.

Previous studies on MDA-MB-468 human breast cancer cells suggested that the expression of MB might correlate with increased cell migration [13]. Thus, we investigated whether MB influences cell migration in MCF7 and SKBR3 cells by using the wound closure assay (Figure 2A,B) and the invasion assay (Figure 2C). Here again, the two cell backgrounds reacted differently upon the loss-of-function (LoF) of MB. While the loss of MB in normoxic MCF7 cells did not alter cell migration, the loss of MB in normoxic SKBR3 cells decreased cell migration (Figure 2B), aligning these findings with previous studies [13]. However, at 0.2% O_2_, MB-KO MCF7 cells migrated almost 2 times faster than WT cells. SKBR3 MB-KO clones differed in cell migration (Figure 2B), thus, yielding ambiguous results. The migration of MB-KO1 cells was unaltered, compared with WT SKBR3, while MB-KO2 cells migrated more slowly than controls. In addition, hypoxic MB-KO MCF7 invaded extracellular-matrix-like substance significantly faster than WT cells (Figure 2D) suggesting that expression of MB helps to prevent cell migration and invasion in this background when O_2_ levels are low.

Since enhanced cell migration, as seen in hypoxic MB-KO MCF7 cells, is frequently associated with mesenchymal features of cells, we analyzed the epithelial to mesenchymal transition (EMT) specifically in this background. EMT is a complex program that incorporates many pathways mediated by the transcription factors SNAIL, SNAI2 (or SLUG), TWIST, and ZEB, whose differential expression in cancer was shown to lead to EMT [25]. Fast migrating hypoxic MB-KO MCF7 indeed showed increased expression levels of EMT markers. The mean expression levels of phospho-Smad 2 and phospho-Smad 1/5/9 in hypoxic MB-KO cells were 2.3 and 2.5 times higher than in WT cells, respectively, while total Smad-2 and E-Cadherin protein levels did not differ (Figure 2E,F). Interestingly, the mean expression level of the p85 regulatory subunit of PI3K, previously shown to promote the motility of MCF7 cells [26], was 2 times higher in hypoxic MB-KO than in hypoxic control WT cells (Figure 2E,F). Furthermore, hypoxic MB-KO MCF7 showed 3 times (*SLUG*), 2 times (*TWIST*), and 2.1 times (*ZEB1*) higher mean mRNA expression levels than hypoxic MB-WT cells (Figure 2G). Mean mRNA levels of fibronectin 1 (*FN1*), a marker associated with an invasive and metastatic breast cancer phenotype and EMT in MCF7 cells [27], were 2.2 times higher in MB-KO cells than in WT cells (Figure 2G). Mean transcript levels of matrix metalloprotease 3 (*MMP-3* or stromelysin 1) in hypoxic MB-KO cells were 2.9 times higher than in hypoxic WT cells (Figure 2G). In contrast, we observed no difference in MMP-9 or MMP-14 expression between hypoxic MB-KO and WT cells (not shown). The transcript levels of subunit B of PI3K, known to be dysregulated in MCF7 and to drive migration of cells [28], were 1.6 times higher in hypoxic MB-KO cells than in hypoxic WT controls (Figure 2G). Our results indicate that many, but not all, EMT markers (e.g., E-Cadherin) are differentially upregulated in hypoxic MB-KO MCF7 cells, implying that MB LoF in this cell background yields at least a partial EMT during hypoxia. 

### 2.3. Endogenously Expressed Myoglobin in BrCa Cells Has No Impact on Cellular Response to Hypoxia

Experimental tumors that overexpressed MB displayed reduced hypoxia with better tumor oxygenation and reduced growth [14]. We investigated whether endogenous low-level MB expression in MCF7 cells also contributes to the cellular response to hypoxia. We observed no differences in expression levels of HIF-1α or HIF-2α proteins on WB (Supplemental Figure S4A) or ICC (Supplemental Figure S4B) when comparing normoxic and 0.2% O_2_-incubated MCF7 cells, with or without MB. We also observed no changes in gene expressions of HIF-1α downstream targets fatty acid synthetase (*FASN*) and *EGLN1*, which encodes the prolyl hydroxylase domain-containing protein 2 (PHD2) (Supplemental Figure S4C). Likewise, no changes were seen in mRNA levels of two downstream targets to HIF-2α, namely, erythropoietin (*EPO*) and *CITED2* (Supplemental Figure S4C). Taken together, the low expression level of MB in MCF7 cells seems incapable of impacting the oxygenation status of the cancer cells as indicated by indirect marker assessments.

### 2.4. Myoglobin Interferes with Estrogen Receptor Expression and ROS Generation

**Figure 3:**
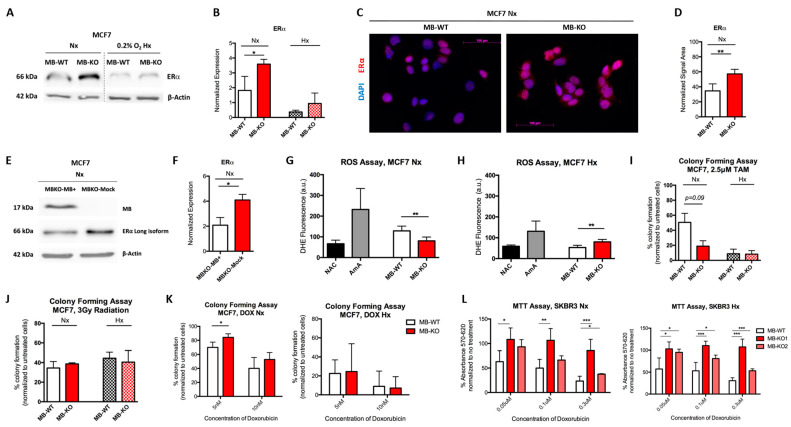
Myoglobin interferes with estrogen receptor expression, ROS generation, and response to chemotherapeutic but not irradiation treatment. (**A**) Representative Western blotting image of whole tissue lysate of MB-WT and MB-KO clones of MCF7 cells cultured at normoxia (Nx) or 0.2% O_2_ hypoxia (Hx) for 72 h and stained for estrogen receptor alpha ERα (60 kDa) and β-actin (42 kDa) used as the loading control (*n* = 4). (**B**) Band intensity of ERα protein after Western blotting, from MB-WT (white) and MB-KO (red) cells of MCF7 at Nx (empty bars) and 0.2%O_2_ Hx (dashed bars), was quantified using MCID Analysis 7.0 and normalized to β-actin (*n* = 4). (**C**) Representative immunocytochemistry images of MB-WT and MB-KO MCF7 cells stained for ERα (red) and DAPI (blue) after culturing at Nx for 72 h. Scale bar is 100 µm. (**D**) Quantitative analysis of ERα signal area as a percentage of the nuclear signal area (*n* = 4). (**E**) Representative Western blotting image of whole tissue lysate of MCF7 MB-KO cells transfected with MB+ or mCherry plasmids and stained for ERα (60kDa) and β-actin (42 kDa) used as the loading control. (**F**) Band intensity of ERα after Western blotting, from MB-KO transfected with MB+ (white) or mCherry plasmids (red) cells at Nx, was quantified using MCID Analysis 7.0 and normalized to β-actin (*n* = 3). (**G**,**H**) Fluorescence assay of dihydroethidium (DHE) to measure ROS directly in living cells cultured at Nx or 0.2% O_2_ Hx for 72 h, respectively. 10,000 cells/well were seeded, in triplicate, in a 96-well plate and incubated at 21% or 0.2% O_2_ for 72 h. Antimycin A (AmA), an inhibitor of complex III of the mitochondrial electron transport chain, was included as a positive control for ROS generation. N-acetylcysteine (NAC) was included as an antioxidant negative control. Fluorescence using an excitation/emission wavelength of 500/590 was used (*n* = 3). (**I**,**J**) Colony assay using MCF7 MB-WT (white) and MB-KO (red) cells seeded at low confluency and treated with 2.5 µM tamoxifen (TAM) (I) or 3Gy irradiation (**J**). Cells were then incubated in Nx (empty bars) or 0.2% O_2_ Hx (dashed bars) for 9 days before colonies (>50 cells) were stained and counted (*n* = 4). Data are presented as % of colony forming normalized to untreated. (**K**) Colony assay using MCF7 MB-WT (white) and MB-KO (red) cells seeded at low confluency and treated with 5 or 10 nM Doxorubicin. Cells were then incubated at Nx or 0.2% O_2_ Hx for 9 days before colonies (>50 cells) were stained and counted (*n* = 4). (**L**) MTT assay using SKBR3 clones. Cells were incubated in Nx (left panel) or 0.2% O_2_ Hx (right panel) with 0.05, 0.1, and 0.3 µM doxorubicin for 72 h. After the addition of MTT, absorbance was measured and normalized to cell number (determined by growth curve experiments) (*n* = 4–6). Data are presented as mean and standard error of mean and analyzed by Student *t*-test except for SKBR3 data in panel (**L**) by One-way ANOVA with Tukey’s multiple comparison post-hoc analysis * *p* < 0.05, ** *p* < 0.01, *** *p* < 0.001.

MB adds to the prognostic value of ERα in human breast cancer patients [8]. Therefore, we analyzed if MB impacts the ERα expression in ER+ MCF7 cells (Figure 3A), but refrained from doing so in ER- SKBR3 cells [29]. The mean expression level of the nuclear long isoform of ERα in normoxic MB-KO cells was 2 times higher than in normoxic WT cells (Figure 3B), which was confirmed by immunofluorescence analyses that showed elevated numbers of ER-positive cells (Figure 3C,D). Re-expression of MB in the normoxic MB-KO cells resulted in downregulation of ERα expression, confirming our earlier observation (Figure 1E,F). Because ERα protein can be destabilized by ROS [30,31], we analyzed ROS levels (primarily superoxide and hydrogen peroxide) in normoxic (Figure 3G) and hypoxic (Figure 3H) MB-KO and WT MCF7 cells, cultured for 72 h. At normoxia, MB-WT cells showed 50% higher mean ROS levels than KO cells (Figure 3G) in the presence of positive (antimycin A: AmA) and negative (N- acetylcysteine: NAC) assay controls. While levels of *SOD3* transcripts in MB-KO MCF7 cells were 10 times lower than in WT cells, other ROS detoxifying genes were not differentially regulated (Supplemental Figure S5). However, this severe downregulation of *SOD3* by the loss of MB suggests the ERα destabilizing ROS in normoxic MB-WT cells to originate at sites outside of the mitochondria. Unsurprisingly, 0.2% O_2_ hypoxia reduced mean ROS levels in WT cells by 2.4-fold relative to their normoxic levels but not in MB-KO cells. MB-deficient MCF7 cells showed elevated ROS levels compared to WT controls under hypoxia (Figure 3H), which might explain the normoxia-specific downregulation of ERα in MB-WT cells relative to MB-KO counterparts.

### 2.5. Myoglobin Interferes with Cancer Cells’ Response to Chemotherapeutic but Not Ionizing Irradiation Treatment

We next tested whether the differential ER expression might influence response to anti-hormone therapy. Indeed, the clonogenic assay revealed a trend towards markedly fewer colonies of MB-KO cells treated with the selective ER modulator tamoxifen (TAM), as compared to WT cells under Nx but not at 0.2% O_2_ Hx (Figure 3I). These results suggest that endogenously expressed MBO_2_ might contribute to regulating ERα expression in oxygenated breast cancer cells. In addition to antihormonal therapy, MB-containing particles were recently targeted toward cultured lung cancer cells, where they improved intracellular O_2_ levels and, thus, sensitized the cells to the radiation therapy [32]. Contrary to the previous findings, our colony formation assays after 9 days revealed no difference in the number of colonies when comparing normoxic or hypoxic MB-KO and MB-WT control cells in response to 3Gy irradiation (Figure 3J). We also analyzed the response of MB-WT and MB-KO cells to doxorubicin (DOX) treatment, which is routinely used in the treatment of several cancers including mammary carcinomas [33]. In normoxia, MB-KO MCF7 cells showed an elevated number of colonies compared to MB-WT cells in response to 5 nM DOX (Figure 3K). A similar, yet non-significant, trend was noted in response to 10 nM of DOX. Under hypoxic conditions, the colony formation of MCF7 WT and MB-KO cells did not differ at low or high concentrations of DOX (Figure 3K), suggesting that only the presence of MBO_2_ sensitizes normoxic MCF7 cells to DOX treatment. Assessing the response of MB-proficient and -deficient SKBR3 cells to DOX was hampered by the complete lack of colony formation by this line. We, thus, needed to use the MTT assay to determine the MB-dependent sensitivity of these cells to DOX. Interestingly, MB-KO SKBR3 cells showed a higher MTT conversion rate than WT cells along with increasing concentrations of doxorubicin under both, normoxic and hypoxic conditions (Figure 3L). Therefore, both oxy and deoxy MB can sensitize SKBR3 cells to DOX treatment. In conclusion, basal MBO_2_ abundance seems to interfere with hormonal receptor expression, perhaps via an intensified ROS generation in oxygenated BrCa cells. The proficiency of MB in MCF7 and SKBR3 backgrounds sensitizes both cell types to treatment with DOX under high O_2_ concentrations, while it renders a TAM-based intervention of ERα-positive normoxic MCF7 cells far less effective. The status of endogenous MB in BrCa backgrounds allegedly has a strong influence on the outcome of different chemotherapeutic interventions. It does not, however, seem to impact the response of the cells to radiation.

### 2.6. Loss of Myoglobin Inhibits Apoptosis in Normoxic BrCa Cells

**Figure 4:**
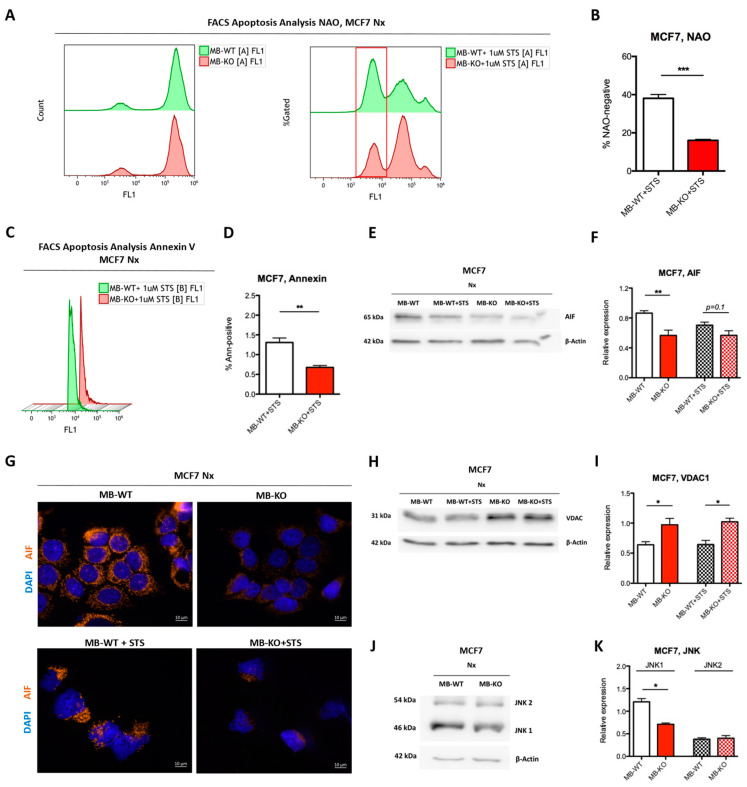
Myoglobin sensitizes breast cancer cells to apoptosis. (**A**) Representative FACS analysis of MB-WT and MB-KO clones of MCF7 cells stained by 10-N-Nonyl acridine orange (NAO), a fluorophore that forms a stable complex with the reduced form of cardiolipin within the inner mitochondrial membrane of living non-apoptotic cells. One hundred thousand cells were incubated, in duplicates, at Nx for 72 h, followed by incubation without (left panel) or with 1 mM of the apoptosis-inducing agent staurosporin (STS) for 6 h (right panel). The cell population with negative NAO fluorescence, marked with a red box, corresponds to the population of apoptotic cells. (**B**) Quantitative analysis of NAO-negative proportion of cells after with 1 mM of STS for 6 h (*n* = 3). (**C**) Representative Annexin V staining-based FACS analysis of MCF7 cells, incubated in duplicates at Nx for 72 h, before treatment with 1 mM of the STS for 6 h, to detect cells at a later stage of apoptosis accompanied by live/dead stain of propidium iodide (PI). (**D**) Quantitative analysis of Annexin V-positive proportion of cells after with 1 mM of STS for 6 h (*n* = 3). (**E**,**H**) Representative Western blotting image of whole tissue lysate of MB-WT and MB-KO clones of MCF7 cells cultured at normoxia (Nx) or 0.2% O_2_ hypoxia (Hx) for 72 h and treated or not with 1 mM STS for 6 h, followed by staining for apoptosis-inducing factor (AIF) (65 kDa) (**E**) or VDAC1 (31 kDa) (**H**) and β-actin (42 kDa) used as the loading control (*n* = 3). (**F**,**I**) Band intensity of AIF and VDAC1 proteins, respectively, after Western blotting, from MB-WT (white) and MB-KO cells (red) of MCF7 cultured at Nx without (empty bars) and with the treatment of 1 mM STS for 6 h (dashed bars), was quantified using MCID Analysis 7.0 and normalized to β-actin (*n* = 3). (**G**) Representative immunocytochemistry images of MB-WT and MB-KO MCF7 cells stained for AIF (orange) and DAPI (blue) after culturing at Nx for 72h (upper panels) or with the treatment of 1 mM STS for 6 h (lower panels). Scale bar is 10 µm. (**J**,**K**) Representative Western blotting image and band intensity analysis of whole tissue lysate of MB-WT (white) and MB-KO (red) clones of MCF7 cells cultured at Nx for 72 h and stained for JNK1 (54 kDa), JNK2 (64 kDa) and β-actin (42 kDa) used as the loading control. Data are presented as the mean and standard error of mean and analyzed by Student’s *t*-test * *p* < 0.05; ** *p* < 0.01; *** *p* < 0.001.

**Figure 5:**
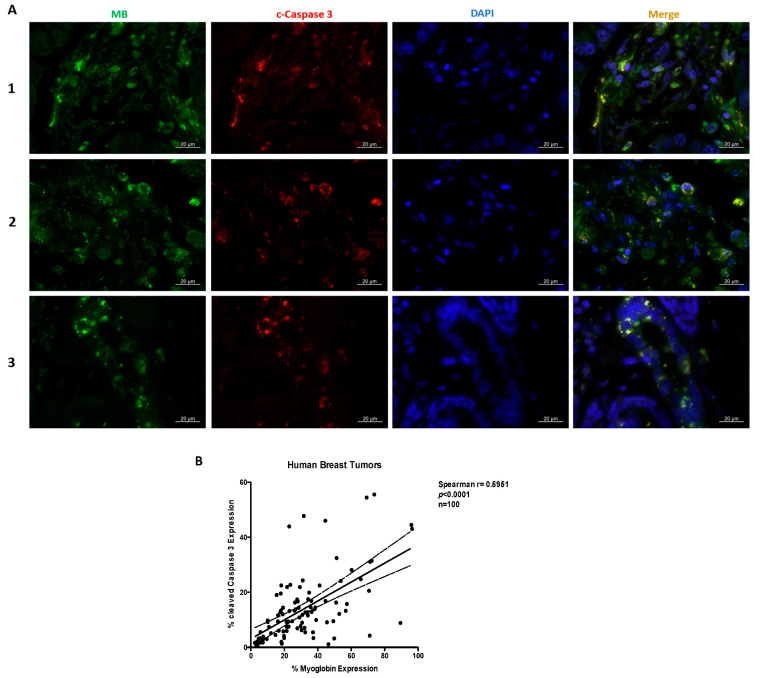
Expression of myoglobin in human invasive ductal carcinoma tumors correlates with increased cleaved caspase 3 expression in vivo. (**A**) Representative immunofluorescence image analysis of the expression of MB (green), cleaved caspase 3 (red), and DAPI (blue) from breast tissue microarray of human invasive ductal carcinoma tumors that are estrogen and progesterone receptors positive. Scale bar is 20 µm. (*n* = 100). (**B**) Correlation analysis of % of stains signal areas normalized to tissue areas (*n* = 100).

Because loss of MB reduced ROS production in oxygenated MCF7 cancer cells and rendered these cells more resistant to DOX treatment (Figure 3G,K), we next focused on the effect of MB-KO on apoptotic stimulation under normoxia. Supplementation of human renal proximal tubule cells with ferrous MB was previously shown to stimulate apoptosis [34]. In addition, the presence of MB also stimulated apoptosis in prostate cancer cells [22]. We induced programmed cell death through incubation with 1µM staurosporin (STS) for 6h and quantified apoptotic cell populations by using flow cytometry with NAO staining (Figure 4A) [35]. The mean number of apoptotic MB-KO MCF7 cells, denoted as completely negative for NAO-based fluorescence, was 2 times lower than in MB-WT cells (Figure 4B), suggesting that MBO_2_ indeed facilitates apoptosis. The NAO-intermediate pool of cells located approximately one log below the positive population (Figure 4A, right panel) was omitted in our quantification of apoptotic cells, as this pool presumably represents cells of mixed oxidized/reduced cardiolipin (CL) composition and/or of the mixed location of CL to inner or outer mitochondrial membrane sites (see NAO-based FACS analysis in Materials and Methods section for more details). In a second apoptosis assay, we looked at the flip of phosphatidylserine to the outer leaflet of the plasma membrane as a late indicator of programmed cell death. Here again, the mean number of Annexin-positive apoptotic cells among the population of MB-KO MCF7 cells was 60% lower than in WT cells, after treatment with 1uM STS for 6h (Figure 4C,D), further supporting that MB-deficiency greatly diminishes the frequency of apoptosis in oxygenated MCF7 cells. Because MCF7 cells do not express caspase-3 [36,37], we analyzed cellular levels of (i) cleaved Lamin A, which is produced by cleaved caspase 6, as well as (ii) cleaved caspase 7. No expression of cleaved Lamin A was detected in MCF7 cells by immunoblotting (also shown by others [38]), nor could we detect a change in protein expression of cleaved caspase 7 in the presence or absence of MB. However, mean levels of apoptosis-inducing factor (AIF) protein, which is a mitochondrial protein that induces caspase-independent apoptosis [39], were reduced by 33% in MB-KO than MB-WT cells (Figure 4E,F). After treatment with STS for 6 h, a smaller nuclear fraction of the AIF protein was associated with the loss of MB as compared with MB-WT cells in conjunction with the weaker overall AIF expression in MB-negative cells (Figure 4G). Additionally, MB-KO MCF7 cells upregulated protein-level expression of the mitochondrial voltage-dependent anion (VDAC1), a protein involved in metabolic apoptosis pathways [40], by 1.6 and 1.5 times relative to MB-WT cells in the presence or absence of 1uM STS for 6h, respectively (Figure 4H,I). Furthermore, the mean expression levels of JNK1, but not JNK2, in normoxic MB-KO cells were 2 times lower than in MB-WT cells (Figure 5J,K) suggesting that lower ROS levels in normoxic MB-KO cells may not be sufficient to activate JNK1-dependent apoptosis [41]. In summary, our data show that MBO_2_ promotes apoptosis in oxygenated MCF7 cancer cells.

To determine whether our in vitro observations regarding apoptosis are also reflected in human cancer tissues in vivo, we measured MB and cleaved caspase 3 expression in tissue arrays from human invasive ductal carcinoma (Figure 5A). The expression of MB in these tumor tissue cohorts correlated positively with the expression of cleaved caspase 3 (Figure 5B). These data further underscore that the presence of MBO_2_ in breast cancer cells and tissues promotes apoptosis.

### 2.7. Differential Gene Expression Analysis by RNA-Seq 

Genes differentially expressed between MB-KO and MB-WT MCF7 single cell clones were analyzed by RNA-Seq after incubation at Nx and Hx (0.2% O_2_) for 72 h. Then, 281 genes were found to be upregulated, and 128 genes were downregulated in response to MB-knockout in normoxic MCF7 cells (*p* adjust ≤ 0.05). Under Hx, 180 genes were upregulated and 81 genes downregulated in MB-KO cells vs WT controls (*p* adjust ≤ 0.05). The overrepresentation analysis using g:Profiler revealed 112 GO-terms of enriched biological processes. Supplemental Figure S6 shows the GO-terms clustered in Cytoscape. In the Nx dataset, overrepresented GO-terms are mainly related to cell adhesion (‘cell adhesion’, ‘biological adhesion’, and ‘cell-cell adhesion via plasma-membrane adhesion molecules’) and cell migration (‘cell migration’). GO-terms for the Hx dataset are mainly related to signaling (e.g., ‘signal transduction’, ‘cell surface receptor signaling pathway’, ‘cell communication’) and to response to different stimuli (e.g., ‘response to external stimulus’, ‘response to organic substance’, ‘response to chemical’). Additional categories are related to cell motility, taxis, and cell adhesion. Furthermore, genes related to ‘cell death’ and ‘programmed cell death’ were also overrepresented by the loss of MB. The terms ‘reactive oxygen species metabolic process’ and ‘regulation of fat cell differentiation’ appeared as well. Full lists of all significantly differentiated genes at Nx and Hx are shown in (Supplemental Tables S1 and S2), including the already mentioned SLUG and SMAD genes (see Figure 2). 

### 2.8. Breast Cancers with Mutant p53 Have Less MB and a Worse Prognosis

**Figure 6:**
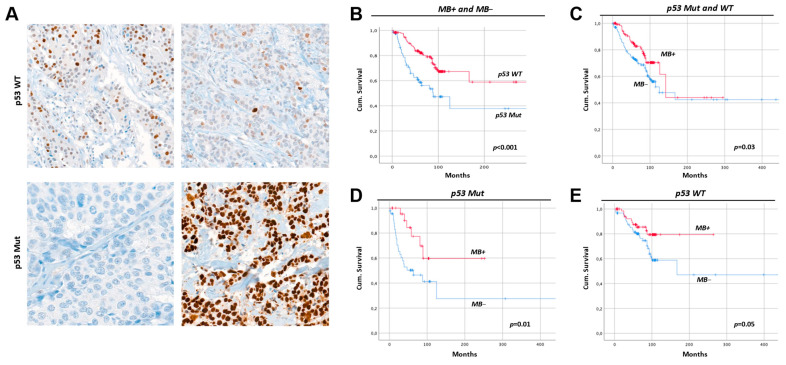
Myoglobin expression is linked to better prognosis independently of p53 status. (**A**) Representative image of immunohistochemistry staining of p53 in a cohort of human invasive ductal carcinoma (*n* = 288). The upper left and upper right panels show moderate and weak expression of wildtype (WT) p53, respectively. The lower left and lower right panels show complete expression loss and pathological accumulation of p53, respectively, displaying the two forms of p53 mutation (p53 Mut) as detected by immunohistochemistry. The blue color is the hematoxylin counterstain of the nucleus, while the brown color is the oxidized product of HRP-DAB indicating p53 stain. Scale bar is 200 mm. (**B**–**E**) Kaplan–Meier analyses of a cohort of 288 primary breast cancer cases displaying (**B**) tumors with p53 WT expression and their significantly improved cumulative overall survival prognosis compared with tumor cases with p53 Mut. (**C**) tumors with high MB expression (MB+) show a significantly improved cumulative overall survival prognosis compared with cases of low Mb expression (MB−) in mixed p53 background status. (**D**,**E**) MB+ tumors show significantly improved cumulative overall survival prognosis compared with MB− tumors, in p53 Mut as well as p53 WT cohorts, respectively.

To investigate a possible correlation between MB and p53 expression, we determined p53 protein expression in tissue arrays from human invasive ductal carcinoma (*n* = 288) that we have analyzed for MB expression in our previous work [8]. The expression of p53 in this tumor tissue cohort was heterogenous, varying between absent, weak, moderate, and strong expression (Figure 6A). At this time, 70.5% of tumors displayed WT p53 expression, while 29.5% exhibited p53 mutation with either complete loss of expression or pathological accumulation of the protein. Patients with p53-WT tumors or MB positivity showed better prognosis and overall cumulative survival as compared to tumors with mutant p53 status or MB negative expression, respectively (Figure 6B,C). MB expression was significantly associated with p53 status: 32.9% of p53-mutant tumors were devoid of MB while only 19.2% of p53-WT showed MB negativity (Table 1). Furthermore, 36.5% of p53 mutant tumors showed moderate to strong MB expression relative to 48.3% of p53-WT tumors. Taken together, p53 WT tumors accumulated more MB protein as compared to p53 mutant tumors. Whether the prognostic value of MB is dependent on p53 status is currently unknown. Nevertheless, MB is associated with a better prognosis in the patients’ group stratified by p53, with a visually more pronounced value in p53-mutant cases (Figure 6D,E).

## 3. Discussion

The most widely recognized roles of MB are the storage and the facilitated delivery of O_2_ to mitochondria in myocytes under hypoxia. However, the very low concentrations at which MB is estimated to be endogenously expressed in cancer cells seem to preclude this function [8]. Here, genetically modified MCF7 breast cancer cells lacking MBO_2_ displayed enhanced survival with upregulated levels of G1/S cell cycle markers. On the other hand, SKBR3 breast cancer cells without MB exhibited reduced survival. When looking deeper into molecular mechanisms in the MCF7 background, we noticed that the loss of MBO_2_ in these cells was associated with fewer ROS being generated in normoxic cells. This resulted in a) upregulated protein levels of ERα, b) a sensitization of cells to tamoxifen-mediated inhibition of colony formation, and the c) desensitization of cells towards chemotherapy-induced apoptosis (e.g., via DOX treatment). Hypoxic MCF7 cells lacking MB showed upregulated EMT markers and increased motility along with increased invasive behavior, hence, a heightened metastatic potential. The lack of MB combined with O_2_ limitation activated SMAD signaling but did not affect the cellular response to irradiation or chemotherapy. The expression of MB in the human tumor tissue cohort correlated positively with the expression of cleaved caspase 3. We report on a novel set of roles and molecular mechanistic insights mediated by basal MB expression in breast cancer cells. The data presented in this study suggest MB contributes to tumor suppression in a subset of breast cancer cells (i.e., p53-WT, ERα-positive) and tumors by reducing ERα expression, cellular migration, as well as cell survival mechanisms. Moreover, our data suggest that non-muscle MB aids in predicting the metastatic behavior of breast cancer patients and, therefore, predicts patients’ outcomes. In addition, MB might serve as a therapeutic target with careful consideration of other factors such as p53 and the hormone receptor status of the tumor. Importantly, our study demonstrates that those roles of MB are operant even with low basal expression levels.

### 3.1. Myoglobin Attenuates Normoxic Breast Cancer Cell Survival and ERα Signaling While Enhancing Apoptosis and Response to Chemotherapy by Increasing ROS Levels

Loss of MB expression correlates with poor patient survival [8,9], suggesting that MB is a tumor suppressor. While supraphysiological levels of MB after ectopic overexpression downregulate cyclin E and reduce the cell cycle progression of cancer cells [42], we show here that endogenous levels of MB in human MCF7 breast cancer cells antagonize cell survival rather than slowing proliferation. We show that the deficiency of the globin increased levels of both cyclin D1, a marker of poor prognosis in breast cancer patients [43], and cyclin E, without increasing cellular proliferation. In contrast, loss of MB in LNCaP prostate cancer cells resulted in low p21 transcripts and increased proliferation [22] suggesting MB to exert tumor-type specific functions. In addition, our previous results in MDA-MB 468 breast cancer cells using si/shRNA MB gene knockdown revealed decreased proliferation in response to MB downregulation. The differences may be explained by different cell line biology or a different knockout mechanism [44]. The induced cyclin D1 levels in our MB-KO cells may also occupy a cell cycle-independent role, e.g., by directly binding to the hormone-binding domain of ERα, mediating gene transcription also in the absence of estrogen [44]. Indeed, we observed induced ERα expression levels and increased cell survival in normoxic MB-KO cells, which might be either explained by the higher cyclin D1 levels and/or by the lower levels of ROS. The elevation of ROS levels can downregulate ERα [30,31,45]. ERα was reported to protect cancer cells from p53-mediated apoptosis induced by DNA damage [46] and from ROS-mediated apoptosis [47]. 

In addition to increasing ERα expression, the loss of MB in normoxic MCF7 cells prevented apoptosis and thus, increased cancer cell survival. We show that AIF levels and JNK activation were reduced in MB-KO cells. In line with this observation, our previous siRNA-based transcriptomic analyses on non-clonal pools of LNCaP prostate cancer cells also reported that the presence of MB in this p53-WT cell line is associated with the stimulation of apoptotic processes [22]. Thus MB`s pro-apoptotic/anti-invasive roles, reported in the current study for the MCF7 clones, clearly occur in a wider range of, presumably p53-WT, epithelial cancer cell backgrounds. The presence of MB in the normoxic cells was associated with elevated ROS levels, along with a very strong compensatory overexpression of the *SOD3* antioxidant gene. This pattern might confine elevated ROS to sites other than mitochondria (i.e., cytosol), which could promote JNK signaling and downstream apoptosis in oxygenated cells [48]. The fact that the presence of MB even promotes the abundance of *SOD3* in hypoxic MCF7 needs to be further explored. We also document a positive correlation between MB and apoptosis protein expression in human invasive ductal carcinoma tissue as proof of concept that our mechanistic findings may hold true in clinical datasets where MB expression is known to correlate with improved outcomes. Indeed, the terms ‘cell death’ and ‘programmed cell death’ were detected in the hypoxia dataset of our transcriptomic analysis. Additionally, we observed increased expression levels of VDAC1 in MB-KO cells. VDAC1, despite its role in mitochondrial metabolism [49], acts as a suppressor of apoptosis by downregulating caspases, p53, cytochrome c, and growth in cancer cells [50] and yeast [51]. Recently, VDAC1 expression was correlated with poor prognosis in human breast [52] and lung [53] cancer patients. In conclusion, our data suggest that the presence of MBO_2_ in oxygenated breast cancer cells renders cancer cells less viable, mainly through its pro-apoptotic functions, and more susceptible to anti-cancer treatments, including Doxorubicin-based interventions.

### 3.2. Myoglobin Regulates the Migratory Capacity of Hypoxic Cancer Cells

To help clarify whether the actual low-level expression of MB detected endogenously in tumors confers meaningful O_2_ storage or buffering capacities [8], we performed our in vitro experiments under normoxic and hypoxic conditions to mimic the oxygenation heterogeneity of clinical tumors and to compare the cellular function of oxygenated (MBO_2_) vs. mainly deoxygenated MB in breast cancer (i.e., applying the Hill equation). Tumor hypoxia has well been documented to correlate with enhanced cancer cell migration. Indeed, hypoxic cells lacking MB showed increased migration and invasion capacity by acquiring some mesenchymal features of the EMT. The loss of MB increased the migration of hypoxic, but not normoxic, MCF7 breast cancer cells through the activation of the SMAD signaling pathway and upregulation of MMP3 expression. Hypoxic MB-KO cells exhibited upregulated phospho-Smad 2 and phospho-Smad 1/5/9 proteins expression as well as upregulation of their downstream targets *TWIST*, *SNAIL*, *SLUG,* and *ZEB1*. This notion is strongly supported by GO-terms related to motility under hypoxia in the RNA-Seq analysis presented in this study. Enrichment analyses in our earlier transcriptomic analysis of three different epithelial cancer cells with basal control (i.e., scrambled RNA) or siRNA-driven reduced MB expression revealed that the GO categories ‘cell motion’ and ‘cell migration’ were significantly overrepresented among the upregulated genes of MB knocked down LNCaP cells compared to WT cells [22]. Hypoxic MB-KO cells showed upregulation of *FN1*, a glycoprotein ECM component of the mesenchymal compartment that is not normally expressed by breast tissue, thus indicating the switching to an increased capacity of invasive and metastatic cancer cell behavior and induced EMT in MCF7 cells [27]. Complementary to that, *MMP3* was differentially upregulated in MB-KO cells, indicating active ECM degradation, which is an important step during tumor metastasis. Since other markers, such as E-Cadherin, did not change their expression level in correlation with the MB status, these data suggest a partial rather than complete switch to an EMT phenotype. Cells revealing a partial EMT were previously shown to be highly enriched in lung metastases, while cells that have undergone a full EMT retain a more quiescent mesenchymal phenotype and do not colonize the lung. In this regard, a partial EMT might very well contribute, or even drive, breast cancer metastasis [54].

### 3.3. Myoglobin in MCF7 vs. SKBR3 and the Hypothesized Interplay with p53

In contrast to MFC7 cells, the loss of MB in SKBR3 breast cancer cells neither influences migration under hypoxia nor the cells’ survival under normoxia (see data summarized in Table 2), suggesting that our findings may not only be specific for different tumor types but also different subtypes of the cancer. MCF7 cells represent the hormone receptor-positive luminal tumor subtype, while SKBR3 cells mirror the HER2-overexpressing entity [55]. Moreover, MCF7 cells express WT p53, while SKBR3 cells express a mutated GoF p53^Arg175His^ variant [56]. Despite the inability to directly drive p53 target gene expression, mutated p53 could function by interacting with other proteins. We previously showed multiple tumor suppressor p63 [57] target genes to be downregulated in MB positive MDA-MB 468 breast cancer cells due to interaction and inactivation of the mutated p53^Arg273His^ leading to enhanced cell survival and decreased apoptosis [22]. In addition, mutated p53 may act as a cofactor of anti-apoptotic genes [58]. MB-mediated activation and stabilization of the p53 mutation via increased NO, ROS, and HIF1α levels may lead to a tumor-promoting effect of MB specifically for GoF p53 expressing cells (MDA-MB 468 and SKBR3) [59]. This might explain the increased survival and migratory capacity of MB-WT SKBR3 cells. Regarding the cells we used herein, the loss of MB increases cell survival in p53-WT MCF7 cells and downregulates apoptosis in MCF7, as it previously diminished the expression of apoptosis-promoting genes in p53-WT LNCaP. Conversely, the impact of MB on cell survival in p53 GoF MDA-MB 468 was inconclusive [22], suggesting MB to play a role in p53-dependent apoptosis. On the other hand, the response to doxorubicin may not depend on the p53 status since MB LoF in both SKBR3 and MCF7 cells increased the resistance to this chemotherapeutic intervention. Together, MB in breast cancer cells emerges to function in p53-dependent and -independent ways.

## 4. Materials and Methods

### 4.1. Cancer Cells Lines and Cell Culture

Human MCF-7, epithelial adenocarcinoma-derived breast cancer cells, were obtained from (ATCC, Manassas, VA, USA, #HTB-22) and cultured in Eagle’s Minimum Essential Medium (EMEM) (ATCC, Manassas, VA, USA, #30-2003) supplemented with 10% heat-inactivated fetal bovine serum (FBS) (Gibco, New York, NY, USA, #10270-106) and 1% Penicillin/Streptomycin (Gibco, New York, NY, USA, #15140-122). Human SKBR3 epithelial adenocarcinoma-derived breast cancer cells were also obtained from (ATCC, Manassas, VA, USA, #HTB-30) and were cultured in Dulbecco’s Modified Eagle Medium (DMEM) (Gibco, New York, NY, USA, #31885023) supplemented with 1% Glutamax (Gibco, New York, NY, USA, #35050061), 10% FBS, and 1% Pen/Strep. Both cell lines are authenticated by ATCC and the short tandem repeat (STR) profile for either one is as follows: MCF7 (Amelogenin:X; CSF1PO:10; D13S317:11; D16S539:11,12; D5S818:11,12; D7S820:8,9; TH01:6; TPOX:9,12; vWA:14,15) and SKBR3 (Amelogenin:X; CSF1PO:12; D13S317:11,12; D16S539:9; D5S818:9,12; D7S820:9,12; TH01:8,9; TPOX:8,11; vWA:17). For passaging and seeding cells, 0.25% Trypsin-EDTA (Gibco, New York, NY, USA, #25200-072) was used to detach the adherent cells after washing with warm phosphate buffer saline (PBS). To specifically study the impact of MB in cells adapted to long-term hypoxia, mimicking tumors, the cells were incubated for 72 h under hypoxic vs. normoxic conditions. As MB can be either oxygenated or deoxygenated, experiments for cell lines were conducted in room air (normoxia, Nx) and 0.2% O_2_ (severe hypoxia) where MB is mostly deoxygenated [60]. Normoxic (21% O_2_) incubation was performed at standard conditions (37 °C, room air with 5% CO_2_) in a Heracell 240 incubator (Heraeus, Hanau, Germany, #H240-CO_2_). Hypoxic incubations were performed in a glove box (Coy Laboratory, Grass Lake, MI, USA, #8302050) at standard 37 °C with 5% CO_2_.

### 4.2. Generation of CRISPR/Cas9-Mediated Knockouts of MB

#### 4.2.1. Preparation of CRISPR Plasmids against Human MB

The online ‘CRISPR Design Tool’, as described by Cong et al., 2015 [61], was used to identify suitable target sites in exon 2 in the human MB gene. Exon 2 of the *MB* gene was selected because it encodes the functionally critical heme pocket residues (E7 distal and F8 proximal histidine residues) [62] (Supplemental Figure S1A). sgRNAs with the lowest rate of off-target effect score were designed with overhangs (indicated as bold in sequences provided in Supplemental Table S3) to facilitate ligation to CRISPR/Cas9 plasmids and ordered from Microsynth (Switzerland). These plasmids are based on all-in-one pSpCas9(BB)-2A-Puro (px459) (Addgene, Watertown, MA, USA, #62988) V2.0 and pSpCas9n(BB)-2A-Puro (px462) V2.0 (Addgene, Watertown, MA, USA, #62987 [63]) that express Cas9 protein from *S. pyogenes* and include the scaffold of the sgRNA [63]. They both contain a selection marker for puromycin and offer bacterial resistance to ampicillin. px459 expresses the wildtype gene for the Cas9 nuclease and, therefore, only one plasmid, containing one sgRNA, is needed to lead Cas9 to the target sequence and introduce a double-strand break. px462, on the other hand, expresses the Cas9n mutant, which does not introduce double strand breaks but cuts one strand of DNA. Here, two plasmids with two different sgRNA are required to guide Cas9n to two different target sites to achieve a double-strand break. sgRNA oligonucleotides were ligated to plasmids using T4 DNA ligase (Promega, Madison, WI, USA, #M1801) and BbsI restriction enzymes (NEB, Ipswich, MA, USA, #R0539L) according to the manufacturer. Ligated plasmids were cloned into 5-a competent *E. coli* (NEB, Ipswich, MA, USA, #C2987I) cultivated on LB-selection plates with ampicillin (1:1000) (Sigma Aldrich, St. Louis, MI, USA). PCR was performed using U6 primer (annealing to vector backbone) and anti-sense sgRNA (annealing to cloned insert) to identify bacterial colonies that successfully incorporated ligated and not empty plasmids. These specific colonies were amplified by culturing in liquid LB-medium with ampicillin (1:1000) followed by plasmid isolation using QIAprep Spin Miniprep Kit (Qiagen, Hilden, Germany, #27104), according to the manufacturer. Isolated CRISPR plasmids were further double-checked for incorporating sgRNAs by (i) sequencing (Microsynth, Balgach, Switzerland) and (ii) failure to linearize upon digestion with BbsI due to lack of the enzyme’s restriction site.

#### 4.2.2. Generation of MB Knockout Clones of MCF7 and SKBR3 Cells

We generated single-cell clones for generating MB knockout cells to exploit cell models with an identical genetic background that only differ in MB expression. The single-cell clones were selected for the CRISPR-knockout approach based on their highest basal as well as the hypoxia-regulated expression of MB (Supplemental Figure S1B,C, red square mark). These clones were further tested for transfectability using pmCherry-N1 control vectors (Takara Bio, Shiga, Japan, #632523). Then, 200,000 cells of selected monocolonies of either cell line were seeded in 6-well plates and transfected on the next day with CRISPR plasmids using Lipofectamine 2000 (Invitrogen by Life Technologies, Carlsbad, CA, USA, #11668019) according to manufacturer instructions. Twenty-four hours post-transfection, cells were selected using 2M puromycin for 48 h for MCF7 and SKBR3. Surviving cells were seeded on single cell bases for screening to identify knockout monoclonal cells. DNA was extracted using homogenization buffer (50 mM KCl, 10 mM Tris/HCl pH 8.3, 10 mM Gelatin, 0.045% NP-40, 0.045% Tween 20) with 50mM proteinase K (Sigma Aldrich, St. Louis, MI, USA, #3115879001). PCR was conducted using primers that flank exon 2 of the human MB gene (Supplemental Table S3), and products were screened by running on 10% PAGE against that of the control wildtype clone. Clones that showed multiple bands, indicating heteroduplexes, were sequenced and further analyzed using the online TIDE tool (https://tide.nki.nl/) (accessed on 1 November 2018). This tool precisely determines the spectrum and frequency of targeted mutations (insertions or deletions) [64]. Candidate clones that showed no trace of a wildtype allele presence were further inspected for separate allelic modifications by cloning purified PCR product into pGEM-T vectors (Promega, Madison, WI, USA, #A1360) and transforming to NEB 5-a competent *E. coli* according to manufacturer instructions. Cloned plasmids were isolated as described above and sequenced (Microsynth, Balgach, Switzerland). DNA sequences were again analyzed by the TIDE tool and aligned to the wildtype MB gene sequence (NCBI nucleotide blast). The resulting modifications in the protein sequence were analyzed with the Expasy Translation Tool (https://web.expasy.org/translate/) (accessed on 7 November 2018) and NCBI protein blast.

### 4.3. Total Protein Extraction and Western Blotting

Cells were lysed in RIPA buffer (50 mM Tris/HCl pH 8, 150 mM NaCl, 1% NP-40, 0.5% Na deoxycholate, 1 mM EDTA, 0.1% SDS) in the presence of Protease Inhibitor Cocktail Set III, EDTA-Free, according to the manufacturer instructions (Merck Millipore, Darmstadt, Germany, #539134). The protein concentration was determined using the Pierce BCA assay (Thermo Scientific, Waltham, MI, USA, #23228, #23224). SDS-PAGE (Bio-Rad, Hercules, CA, USA) was used for the separation of proteins, which were transferred to nitrocellulose blotting membrane (GE Healthcare, Chicago, IL, USA, #10600002). Following washing with 0.05% Tris-buffered saline-Tween (TBST), membranes were blocked in 5% skimmed milk (or 5% FBS for phospho-proteins) in 0.05% TBST for 1 h at room temp. Next, membranes were incubated at 4 °C overnight with following antibodies: rabbit anti-myoglobin (FL-154) polyclonal antibody (Santa Cruz, Dallas, TX, USA, #sc-25607) 1:200; mouse anti-p53 (DO-1) monoclonal antibody (Santa Cruz, Dallas, TX, USA, #sc-126) 1:200; mouse anti-cyclin D1 monoclonal antibody (Santa Cruz, Dallas, TX, USA, #sc-8396) 1:200; mouse anti-cyclin E (HE12) monoclonal antibody (Santa Cruz, Dallas, TX, USA, #sc-247) 1:200; mouse anti-Rb (IF8) monoclonal antibody (Santa Cruz, Dallas, TX, USA, #sc-102) 1:200; mouse anti-Phospho-Rb (B-4) monoclonal antibody (Santa Cruz, Dallas, TX, USA, #sc-514031) 1:200; mouse anti-ERα (F-10) monoclonal antibody (Santa Cruz, Dallas, TX, USA, #sc-8002) 1:200; rabbit anti-PI3K p85 polyclonal antibody (Cell Signaling, Danvers, MA, USA, #4292) 1:1000; rabbit anti-AKT polyclonal antibody (Cell Signaling, Danvers, MA, USA, #9272) 1:1000; rabbit anti-Phospho-AKT (Ser473) polyclonal antibody (Cell Signaling, Danvers, MA, USA, #9271) 1:1000; rabbit anti-Smad2/3 polyclonal antibody (Cell Signaling, Danvers, MA, USA, #5678) 1:1000; rabbit anti-Phospho-Smad2 (Ser245/250/255) polyclonal antibody (Cell Signaling, Danvers, MA, USA, #3104) 1:1000; rabbit anti-Phospho-Smad1 (Ser463/465)/Smad5 (Ser463/465)/Smad9 (Ser465/467) (D5B10) monoclonal antibody (Cell Signaling, Danvers, MA, USA, #13820) 1:1000; mouse anti-E-Cadherin (36) monoclonal antibody (BD Biosciences, New Jersey, USA, #610182) 1:5000; mouse anti-HIF-1α (mgc3) monoclonal antibody (Abcam, Cambridge, UK, #ab16066) 1:1000; rabbit anti-HIF-2α (EPAS1) polyclonal antibody (Novus, Centennial, CO, USA, #NB100-122) 1:1000; rabbit anti-AIF polyclonal antibody (Abcam, Cambridge, UK, #ab1998) 1:1000; rabbit anti-VDAC1/Porin polyclonal antibody (Abcam, Cambridge, UK, #ab15895) 1:1000; rabbit anti-JNK Pan Specific polyclonal antibody (R&D, #AF1387) 0.2 ug/mL; mouse anti-ATG5 (7C6) monoclonal antibody (nanoTools, Teningen, Germany, #0262) 0.5 ug/mL; mouse anti-LC3 (5F10) monoclonal antibody (nanoTools, Teningen, Germany, #0231) 0.5 ug/mL; mouse anti-p27 (F-8) monoclonal antibody (Santa Cruz, Dallas, TX, USA, #sc-1641) 1:200; mouse anti-p21 (F-5) monoclonal antibody (Santa Cruz, Dallas, TX, USA, #sc-6246) 1:200; mouse anti-cyclin A (H-3) monoclonal antibody (Santa Cruz, Dallas, TX, USA, #sc-271645) 1:100; mouse anti-PCNA (PC10) monoclonal antibody (Santa Cruz, Dallas, TX, USA, #sc-56) 1:200; mouse anti-beta-actin monoclonal antibody (Sigma Aldrich, St. Louis, MI, USA, #A5441) 1:5000. After washing, membranes were incubated with HRP-conjugated secondary antibodies: donkey-anti-rabbit 1:5000 (Amersham, Buckinghamshire, UK, #NA934V) or goat-anti-mouse 1:5000 (Santa Cruz, Dallas, TX, USA, #sc-2031), for 1h at room temp. After washing, bands were visualized by Fujifilm LAS-3000 Chemiluminescent imager using SuperSignal^TM^ West Femto (Thermo Scientific, Waltham, MI, USA, #34095). For quantification purposes, the band peak intensity was analyzed by MCID Analysis 7.0 software.

### 4.4. RNA Extraction and Real-Time PCR

Cultured cells were washed twice with 1x PBS followed by lysis and RNA extraction using ReliaPrep RNA Cell Miniprep System (Promega, Madison, WI, USA, #Z6011) according to the manufacturer. First-strand cDNA was synthesized following the manufacturer’s protocol using RevertAid First Strand cDNA Synthesis Kit (Thermo Scientific, Waltham, MI, USA, #K1622). A final cDNA concentration of 5ng/µL was used for semi-quantitative real-time PCR analysis performed in Thermocycler ABI7500 Fast (Applied Biosystems) using PowerUp SYBR Green Master Mix (Applied Biosystems, Waltham, MI, USA, #A25778). Primers were designed with Primer3 and are listed in (Supplemental Table S3). Primers were validated first by qRT-PCR via i) melting curve analyses (mode integrated into the 7500 Fast Real-Time PCR System to confirm having one single peak corresponding to desired product) as well as ii) on acrylamide gels to confirm the size and purity of PCR products iii) Sanger sequencing of some of the PCR products. The DDCt method was used to calculate mRNA expression levels [65,66].

### 4.5. Trypan Blue Viability Assay

In a 6-well plate, 10,000 cells were seeded in duplicates using a 10% FBS-containing culture medium. The next morning, cells were incubated at 21 or 0.2% O_2_. From day 3 to 8, cells were trypsinized, then mixed with 0.4% trypan blue solution in PBS (Sigma Aldrich St. Louis, MI, USA, #T6146) in a 1:1 ratio and counted by hemocytometer (Neubau Chamber). Only viable cells (white) were counted and plotted as a growth curve over a period of 9 days, while dead cells (blue) were excluded.

### 4.6. Colony Forming Assay

The clonogenic assay was used to evaluate reproductive viability and survival of cells, so the mitotic capacity of a single cell under different conditions and treatments was assessed [67]. First, 250 cells were seeded in a 20% FBS containing culture medium in either a 6-well plate (for Dox or TAM treatment) or T25 angled neck culture flask with vent/close cap (TPP, Trasadingen, Switzerland, #90025) (for radiation treatment). The next morning, 5 nM or 10 nM of doxorubicin as well as 2.5 mM of tamoxifen were added, and cells were incubated at 21 or 0.2% O_2_ environments for 9 days. Solvent-treated cells were used as a control. After overnight incubation, caps were closed, and all flasks including untreated controls were transferred to the irradiation facility. Normoxic or hypoxic cells designated for irradiation were treated with 3Gy of radiation. Flasks were moved back to 21 or 0.2% O_2_ incubators where they were vented and incubated for 9 days. The formed colonies were washed and then fixed/stained using a mixture of 0.5% crystal violet and 6% glutaraldehyde overnight at room temperature. Plates or flasks were then washed with water several times and then air dried. Counted colonies were defined as aggregates of at least 50 cells. The number of colonies was normalized to that of untreated controls.

### 4.7. BrdU Proliferation Assay

The BrdU assay was performed using the Cell Proliferation ELISA BrdU assay (Roche, Basel, Switzerland, #11647229001) according to the manufacturer. Briefly, 20,000 cells/well were cultured in triplicate in 96-well plates using a complete culture medium and incubated for 72 h at 21% normoxic or 0.2% O_2_ hypoxic environments. After incubation, 10 mM of BrdU labeling solution was added, and cells were incubated for 4.5 h at 37 °C. The labeling medium was removed, and cells were fixed, followed by staining with an anti-BrdU-POD working solution for 90 min at room temperature. Wells were rinsed three times with 1X PBS followed by color development by adding substrate and 1 M H_2_SO_4_ stop solution. Photometric detection was carried out within 5 min of adding stop solution at 450 nm (reference wavelength is 690 nm) by Multiskan RC plate reader (Thermo Labsystems, Helsinki, Finland).

### 4.8. MTT Assay

The MTT (Methyl thiazolyl diphenyl-tetrazolium bromide) reduction assay combines the measurement of cell proliferation with the assessment of mitochondria activity. Mitochondrial dehydrogenases of only viable cells will convert MTT to formazan products within the mitochondria. First, 20,000 cells/well were cultured in triplicates in a 96-well plate and incubated overnight. Cells were incubated at 21 or 0.2% O_2_ with or without (0.05, 0.1 and 0.3 uM) doxorubicin (Dox) for 72 h. Then, 500 mM of freshly prepared MTT reagent (Sigma Aldrich, St. Louis, MI, USA, #M5655) was added to each well and incubated for 4 h at 37 °C in the dark. The medium was removed and 100µL of dimethyl sulfoxide (DMSO) (Sigma Aldrich, St. Louis, MI, USA) solvent was added to dissolve the purple precipitate of formazan. Absorbance was measured at 570 nm (reference wavelength is 620 nm) by a Multiskan RC plate reader (Thermo Labsystems, Helsinki, Finland).

### 4.9. Migration Assay

The migratory capacity of cancer cells was assessed by scratch assay. In 6-well plates, 1.2 million cells were cultured per well in duplicate in serum-reduced medium to minimize proliferation and, hence, observe migration only. After overnight incubation, a wound was swiftly created into the cell’s confluent monolayer and cells were incubated at 21 or 0.2% O_2_ for 48 h. Pictures were taken from the same 3 spots (predefined by reference lines drawn on the bottom of the plates) at 0, 24, and 48 h from wound generation. Images were captured using an inverted Axiocam HR microscope coupled with a CCD camera (10x magnification) and analyzed by ImageJ software (version 1.51). The area of the wound was measured and the average of the 3 images at each time point was calculated. A scatter plot was created as % of normalized wound area as a function of time of wound generation.

### 4.10. Cell Cycle Status Analysis

The protocol was adapted from Kim and Sederstrom [68]. It differentiates resting (G0) from proliferating cells by determining the total RNA content of cells, as G0 cells have much lower RNA levels compared to (G1-S-G2-M) cells. In the presence of Hoechst 33342, which stains double-stranded DNA, and Pyronin Y, which exclusively stains RNA, both signals were quantified by flow cytometry (FACS) analysis. Briefly, 1 million cells were incubated in 10-cm plates in duplicate overnight in a serum-free culture medium to align cells’ cycle status. After 72 h of incubation in a 10% FBS culture medium, cells were harvested, washed, and fixed by adding pre-chilled 70% ethanol dropwise while vortexing, followed by 2 h incubation at −20. Following multiple cycles of washing and centrifuging, cells were stained using 2mM Hoechst 33342 (Sigma Aldrich, St. Louis, MI, USA, #14533) and 4mM Pyronin Y (Sigma Aldrich, St. Louis, MI, USA, #83200) double staining solution. Samples were incubated in the dark for 20 min followed by fluorescence analysis by Gallios Flow Cytometer 561 ready (Beckman Coulter, Brea, CA, USA). UV (355 nm) and blue (488 nm) lasers were used for excitation as well as the 461 nm and 575 nm filter set for Hoechst 33342 and Pyronin Y, respectively.

### 4.11. Annexin-V and NAO-Based FACS Analysis of Apoptosis

A key event in the development of apoptosis is the oxidation of the mitochondrial inner membrane key lipid cardiolipin (CL), followed by the lipids externalization to the outer mitochondrial membrane (OMM), which is subsequently followed by the release of cytochrome c from the mitochondria into the cytoplasm. 10-N-Nonyl acridine orange (NAO), a fluorophore that forms a stable complex selectively with the reduced form of CL, but not with oxidized CL, can be used for flow cytometry analysis of the extent of the surface of the inner mitochondrial membrane (IMM) [35]. Therefore, cell populations with negative NAO fluorescence correspond to the population of apoptotic cells, i.e., those cells whose pool of CL exists as the oxidized lipid in the OMM [69]. As a second apoptosis assay, we used the conventional Annexin V staining, which binds to phosphatidylserine exposed in the outer membrane that precedes the loss of membrane integrity that accompanies the later stages of cell death. The stain was either accompanied by a live/dead stain of propidium iodide (PI). First, 100,000 cells were incubated in duplicate at 21% O_2_ for 72 h, followed by incubation with 1 mM of the apoptosis-inducing agent staurosporin (STS) for 6 h. Cells were harvested, washed, and stained for either 100 nM NAO (Sigma Aldrich, St. Louis, MI, USA) (for 30 min in dark at 37 °C) or Annexin V-FITC (Promokine, Heidelberg, Germany, #PK-CA577-K101-100) (5 mL for 5 min in dark at room temperature), followed by fluorescence analysis (Ex = 488 nm; Em = 530 nm) using Gallios Flow Cytometer 561 ready (Beckman Coulter, Brea, CA, USA).

### 4.12. Invasion Assay

The assay protocol for MCF7 cells was adapted from [70]. First, 50 mL of Matrigel basement membrane matrix (Qiagen, Hilden, Germany, #354234) prepared in serum-free culture medium (1:8 ratio to reach a final concentration of 1.2 mg/mL) was used to coat 8µm PET membrane Transwell inserts in 24-well plates (Corning, New York, NY, USA, #3464) to mimic the extracellular matrix. The Transwell inserts were incubated with Matrigel at 37 °C overnight to solidify. Then, 100,000 cells in reduced serum culture medium were placed in the upper chamber and incubated for 48 h at 21% or 0.2% O_2_, with 10% serum medium placed in the lower chamber as a chemoattractant. The inserts were then washed twice with 1X PBS, then cells were fixed and stained simultaneously in a mixture of 0.5% crystal violet and 6% glutaraldehyde overnight at room temperature. The inserts were washed with water several times and then air dried. Cells on the upper surface of the membrane (non-invading cells) were removed by moistened cotton swab and invading cells existing on the lower surface were imaged by Digital Microscope VHX-6000 (Keyence, Osaka, Japan). Five random pictures were captured from each insert and the crystal violet signal was quantified using ImageJ software (version 1.51).

### 4.13. ROS Measurement

The DHE (Dihydroethidium) assay kit (Abcam, Cambridge, UK, #ab236206) was used to measure ROS directly in living cells. The kit is specific for superoxide and hydrogen peroxide and was used according to the manufacturer’s instructions. First, 10,000 cells/well were seeded in triplicate in a 96-well plate and incubated at 21 or 0.2% O_2_ for 72 h. Antimycin A, an inhibitor of complex III of the mitochondrial electron transport chain, was included as a positive control for ROS generation. N-acetyl Cysteine was included as an antioxidant negative control. Fluorescence using excitation/emission wavelength of 500/590 was measured by Infinite 200 Pro fluorescent multi-chromate plate reader (Tecan, Männedorf, Switzerland).

### 4.14. Immunofluorescence

First, 50,000 cells were cultured on coverslips placed in 24-well plates and incubated at standard culture conditions overnight. After attaching, plates were incubated at 21 or 0.2% O_2_ for 72 h. Cells were fixed in 4% paraformaldehyde then washed and permeabilized in 0.1% triton/PBS followed by washing. Cells were then blocked in 10% normal goat serum (NGS) in PBS for 1h at room temperature followed by staining with: mouse anti-ERα (F-10) monoclonal antibody (Santa Cruz, Dallas, TX, USA, #sc-8002) 1:50; mouse anti-HIF-1α (mgc3) monoclonal antibody (Abcam, Cambridge, UK, #ab16066) 1:200; rabbit anti-HIF-2α (EPAS1) polyclonal antibody (Novus, Centennial, CO, USA, #NB100-122) 1:200; rabbit anti-AIF polyclonal antibody (Abcam, Cambridge, UK, #ab1998) 1ug/mL, antibodies in 3% NGS in PBS overnight at 4 °C in wet chamber. Coverslips were then washed in PBS-Tween and incubated with Alexa 546-a-mouse secondary antibody (Thermo Fisher, Waltham, MI, USA) 1:500 in 3% NGS in PBS and counterstained by DAPI (Sigma Aldrich, St. Louis, MI, USA) 1:1000 in PBS for 1h at room temperature. Slides were mounted using Fluorescent Mounting Medium (Dako, Santa Clara, CA, USA, #S3023), sealed with nail polish, and kept at 4 °C in the dark. Images were then taken using a Zeiss Imager Z2 fluorescent microscope coupled with a CCD camera.

### 4.15. Myoglobin Expression

RNA from MDA-MB468 cells was extracted with the RNeasy mini kit (Qiagen, Hilden, Germany) and converted to cDNA with Superscript III (Thermo Scientific, Waltham, MI, USA). The CDS of the MB gene was amplified with the KAPA HiFi PCR Kit (Peqlab) and flanked with restriction enzyme target sites. PCR products were cleaned with the Wizard SV Gel and PCR Clean-Up Kit (Promega, Madison, WI, USA) and cut with XhoI and BamHI or HindIII and BamHI (Thermo Scientific, Waltham, MI, USA). After vector dephosphorylation, MB CDS were ligated in XhoI and BamHI digested pmCherry-C1 (Takara Bio, Shiga, Japan, #632524) or in HindIII and BamHI digested pmCherry-N1 (Takara Bio, Shiga, Japan, #632523) with T4 DNA ligase (Thermo Scientific, Waltham, MI, USA). Plasmids were transformed into DH10B cells by electroporation and plated on 50 mg/µL Kanamycin agar. Single clones were amplified in LB media, isolated with the Gene JET Plasmid Miniprep Kit (Thermo Scientific, Waltham, MI, USA) and approved by Sanger sequencing (Starseq). For overexpression experiments, cells were seeded in 12-well plates to reach 70% confluency on the next day. For each well, 3.3 μg DNA of either plasmid was added to 150 μL Opti-MEM reduced serum media (Gibco, New York, NY, USA, #31985062) and then mixed thoroughly with 9.9 μL Fugene HD transfection reagent (Promega, Madison, WI, USA, #E2311). After 10 min, 150 μL of the complex was added to each well and left for 6 h before changing the medium to a complete growth medium and incubation at 21, 1, and 0.2% O_2_ for 48 h. Whole-cell lysates and RNA were subsequently isolated from all clones. Pictures were taken on days 1 and 2 to confirm successful transfection.

### 4.16. RNA Sequencing

#### 4.16.1. RNA Quality Control and Sequencing

RNA was quality-checked on an Agilent RNA nano bioanalyzer, yielding RNA integrity numbers between 9.6 and 10. RNA was quantified with the Qubit RNA BR Assay (Thermo Fischer, Waltham, MA, USA). Illumina sequencing libraries were generated with the NEB Next Ultra II directional RNA library prep Illumina kit. Then, 50 bp single-end Illumina sequencing was performed on an Illumina HiSeq2500 sequencing platform (NGS core facility, University Mainz, Mainz, Germany). Before RNA-Seq, clone authenticity was approved by Sanger Sequencing (Starseq, Mainz, Germany).

#### 4.16.2. Mapping against the Reference Genome

RNA-Seq data were filtered for adapter sequences and trimmed to a quality score greater than 20 with the program package bbduk (BBTools suite; https://sourceforge.net/projects/bbmap/) (accessed on 8 October 2019). Reads shorter than 20 bp were discarded. The genome index file was created in STAR version 2.7.3a [71] with a sjdbOverhang of 38 using the aforementioned reference GRCh38 and the annotation release 98 (http://ftp.ensembl.org/pub/release-98/gtf/homo_sapiens/Homo_sapiens.GRCh38.98.gtf.gz) (accessed on 11 October 2019). The cleaned reads were mapped with the STAR aligner version 2.7.3a [71] to the human genome GRCh38, downloaded from ENSEMBL (ftp://ftp.ensembl.org/pub/current_fasta/homo_sapiens/dna/ftp://ftp.ensembl.org/pub/current_fasta/homo_sapiens/dna/Homo_sapiens.GRCh38.dna.primary_assembly.fa.gz) (accessed on 11 October 2019). A multimap filter was applied with a cut-off of 20; the maximum mismatch allowance was set to 0.04. The minimum overhang for non-annotated splice junctions was set to 8, and the minimum overhang for annotated splice junctions was set to 1. Count data files were generated from each mapping to determine differential gene expression between samples with DeSeq2, which is part of the Bioconductor package for R [72]. All R analyses were performed in RStudio Version 1.0.153. Values were normalized using the rlog function and converted to log2 values.

#### 4.16.3. Gene Ontology Analyses

The Webtool g:Profiler [73] and corresponding Ensembl IDs were applied for the over-representation analysis (ORA) of differentially expressed genes (DEGs) with an adjusted *p*-value < 0.05. The analysis was performed using the gene ontology (GO) biological process and KEGG pathways database [74], yielding a generic enrichment map and a gene matrix transposed file. Both were processed with the EnrichmentMap Cytoscape App 3.3.1 in Cytoscape 3.8.2. The network was created with an edge Cutoff at a Jaccard-Index of 0.25. The resulting network was MCL clustered with clusterMaker2 Cytoscape App 1.3.1 with the similarity coefficient for edge weight and annotated with the AutoAnnotate Cytoscape App 1.3.3. For clustering, part of the CoSE (compound spring embedded) was chosen as the layout algorithm, and the network was scaled for better distribution. For an additional visualization of the ORA results, the enrichment fold change was calculated using the GMT file downloaded from the g:Profiler website as the background list. The percentage of differentially expressed genes (DEGs) associated with a specific term was divided by the percentage of background list genes with the same term. The results were plotted in R using ggplot2. RNA-Seq data will be available at EBI, project number PRJEB51094.

### 4.17. Human Cancer Tissue Array Staining

The breast cancer array (BC081120d) was purchased from US Biomax, Inc. (Derwood, MD, USA), and was subjected to antigen retrieval at 98 °C for 20 min in EDTA buffer (pH 9.0) in a steamer and incubated with a rabbit anti-cleaved caspase 3 antibody (Cell Signaling, Danvers, MA, USA, #9664L) 1:150 overnight at 4 °C followed by anti-rabbit 594 (Invitrogen, Waltham, MA, USA, #A11012) 1:400 for 1h at room temp. Subsequently, the array was incubated with a monoclonal rabbit anti-MB (Abcam, Cambridge, UK, #ab77232) 1:400 for 1h at room temp. followed by anti-Rb 488 (Invitrogen, #A11008) 1:500 for 1 h at room temp. and DAPI for 15 min. Slides were fully scanned (NanoZoomer 2.0-HT; Hamamatsu, Hamamatsu City, Japan) and images of individual cores were captured. For immunohistochemical analysis of p53, our study included tissue microarrays of invasive breast cancer patients diagnosed at the Institute of Surgical Pathology (University Hospital, Zurich, Switzerland), as described [8]. Tissue sections were processed using automated immunohistochemistry platforms (BOND, Labvision (Fremont, CA, USA) and Benchmark, Ventana (Tucson, AZ, USA)) using anti-p53 (DO7) antibody and scanned for further evaluation.

## 5. Conclusions

Our study established that endogenously expressed MB contributes to tumor suppression mechanisms in breast cancer cells and some developing tumors in ways depending on the cell’s oxygenation status. Normoxic breast cancer cells lacking MBO_2_ show enhanced survival and more resistance to chemotherapy-induced apoptosis, while MB-deficient hypoxic cancer cells display augmented migratory and invasive phenotypes. Our findings suggest MB contributes to tumor suppression in a subset of breast cancer cells by regulating cell cycle markers, ROS generation, and hormone receptor expression, controlling cellular motility as well as cancer cell survival and death mechanisms. Moreover, our data suggest that non-muscle MB aids in predicting the metastatic behavior and chemotherapeutic response of breast cancer patients and, therefore, predicting patient prognosis.

## Figures and Tables

**Table 1:** Correlation of myoglobin (MB) expression to p53 status.

p53 Status	MB Expression n (%)					*p*
	No	Mild	Moderate	Strong	Total	
Mutant	28 (32.9)	26 (30.6)	23 (27.1)	8 (9.4)	85 (100)	0.017
Wildtype	39 (19.2)	66 (32.5)	70 (34.5)	28 (13.8)	203 (100)	
Total	67 (23.3)	92 (31.9)	93 (32.3)	36 (12.5)	288 (100)	

**Table 2:** Effect of MB expression in MCF7 and SKBR3 cells as compared to MB-KO cells.

Effect (O_2_)	MCF7	SKBR3
Growth curve (Nx)	🡇	🡅
Migration (Hx)	🡇	inconclusive
Response to Doxorubicin (Nx)	🡅	🡅
Hormonal receptors [55]	ER+ PR+	ER− PR−
p53 status [56]	WT	GoF Mutation
Tumor subtype [55]	Luminal A	HER2 overexpressed

## Data Availability

RNA-Seq data will be available at EBI, project number PRJEB51094.

## Supplementary Materials

The following supporting information can be downloaded at: https://www.mdpi.com/article/10.3390/ijms231911483/s1.
