# Supplementary material for: Pro-Apoptotic and Anti-Invasive Properties Underscore the Tumor-Suppressing Impact of Myoglobin on a Subset of Human Breast Cancer Cells
Source: Int J Mol Sci. 2022 Sep 29;23(19):11483. doi: 10.3390/ijms231911483 (PMC9570501; doi:10.3390/ijms231911483)
Supplement: Supplementary file 1 [file ijms-23-11483-s001.zip › ijms-1816876-supplementary.pdf]

Supplementary Figures

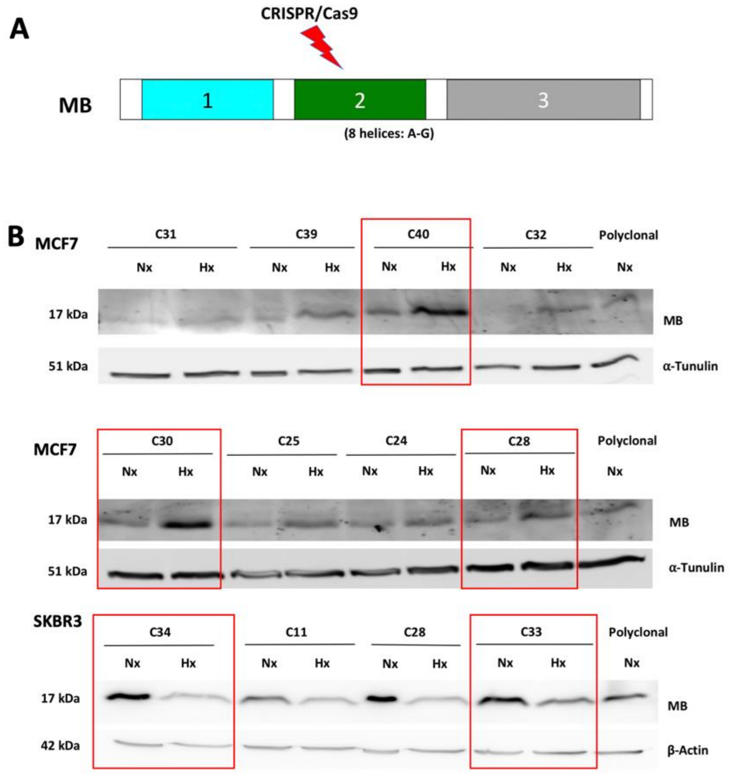

Supplemental Figure S1. Generation of MB knockout clones from MCF7 and SKBR3 cells
(A) Representation of human *MB* gene composed of 3 exons. Exon 2 was targeted to knockout the gene by CRISPR/Cas9 to obtain loss of function. **(B)** Representative Western blot to examine basal MB expression at normoxia (Nx) and (Hx, 0.2% O_2) in different monoclonal cell lines (denoted as C: clone and number) produced from the paternal MCF7 or SKBR3.

A

WT human MB exon 2 DNA sequence
5`..GCTCTTTAAGGGTCACCCAGAGACTCTGGAGAAGTTTGACAAGTTCAAGCACCTGAAGTCAGAGGACGAGATGAAGGCGTCTGAGGACTTAAAGAAGCATGGTGCCACCGTGCTCACCGCCCTGGGTGGCAT
CCTTAAGAAGAAGGGGCATCATGAGGCAGAGATTAAGCCCCTGGCACAGTCGCATGCCACCAAGCACAAGATCCCCGTGAAGTACCT.. 3`

MB exon 2 DNA sequence of MCF7 MBko
Modified Allele 1
5`..GCTCTTTAAGGGTCACCCAGAGACTCTGGAGAAGTTTGACAAGTTCAAGCACCTGAAGTCAGAGGACGAGATGAAGGCATATCTGAGGACTTAAAGAAGCATGGTGCCACTGTGCTCACCGCCCTGGGTGGCA
TCCTTAAGAAGAAGGGGCATCATGAGGCAGAGATTAAGCCCCTGGCACAGTCGCATGCCACCAAGCACAAGATCCCCGTGAAGTACCT..3` 1bp change/+2bp insertion= **+2bp** TAA: premature stop codon
Modified Allele 2
5`..GCTCTTTAAGGGTCACCCAGAGACTCTGGAGAAGTTTGACAAGTTCAAGCACCTGAAGTCAGAGGACGAGATGAAGGCATGACTTAAAGAAGCATGGTGCCACCGTGCTCACCGCCCTGGGTGGCATCCTTAA
GAAGAAGGGGCATCATGAGGCAGAGATTAAGCCCCTGGCACAGTCGCATGCCACCAAGCACAAGATCCCCGTGAAGTACCT.. 3` 1bp change/-5bp deletion= **-5bp** TGA: premature stop codon

WT human MB protein sequence
MGLSDGEWQLVLNVWGKVEADIPGHGQEVLIRLFKGHPETLEKFDKFKHLKSEDEMKASEDLKKHGATVLTALGGILKKKGHHEAEIKPLAQSHATKHKIPVKYLEFISECIIQVLQSKHPGDFGADAQGAMNKALELFRKD
MASNYKELGFQG
154 amino acids, exon 2, H proximal and distal histidines

MB protein sequence of MCF7 MBko
Modified allele 1
MGLSDGEWQLVLNVWGKVEADIPGHGQEVLIRLFKGHPETLEKFDKFKHLKSEDEMKAYLRT truncated with lack of proximal and distal histidines → **Loss of function**
Modified allele 2
MGLSDGEWQLVLNVWGKVEADIPGHGQEVLIRLFKGHPETLEKFDKFKHLKSEDEMKA truncated with lack of proximal and distal histidines → **Loss of function**

B

MB exon 2 DNA sequence of SKBR3 MBko1
Modified Allele 1
5`..GCTCTTTAAGGGTCACCCAGAGACTCTGGAGAAGTTTGACAAGTTCAAGCACCTGAAGTCAGAGGACGAGATGAAGGCGTCTGAGGACTTAAAGAAGCATGGTGCGATGAAGGCATCTGAGGACTTAAAGAA
GCATGGTCCGCCCTGGGTGGCATCCTTAAGAAGAAGGGGCATCATGAGGCAGAGATTAAGCCCCTGGCACAGTCGCATGCCACCAAGCACAAGATCCCCGTGAAGTACCT..3` +14bp insertion= **+14bp** TGG:
premature stop codon
Modified Allele 2
5`..CTCTTTAAGGGTCACCCAGAGACTCTGGAGAAGTTTGACAAGTTCAAGCACCTGAAGTCAGAGGACGAGATGAAGGCGTCTGAGGACTTAAAGAAGCATGGTCCGCCCTGGGTGGCATCCTTAAGAAGAAGG
GGCATCATGAGGCAGAGATTAAGCCCCTGGCACAGTCGCATGCCACCAAGCACAAGATCCCCGTGAAGTACCT.. 3` -13bp insertion= **-13bp** TGA: premature stop codon

MB protein sequence of SKBR3 MBko1
Modified allele 1
MGLSDGEWQLVLNVWGKVEADIPGHGQEVLIRLFKGHPETLEKFDKFKHLKSEDEMKASEDLKKHG truncated with lack of distal histidine → **Loss of function**
Modified allele 2
MGLSDGEWQLVLNVWGKVEADIPGHGQEVLIRLFKGHPETLEKFDKFKHLKSEDEMKASEDLKKHG truncated with lack of distal histidine → **Loss of function**

C

MB exon 2 DNA sequence of SKBR3 MBko2
Modified Allele 1
5`..CTCTTTAAGGGTCACCCAGAGACTCTGGAGAAGTTTGACAAGTTCAAGCACCTGAAGTCAGAGGACGAGATGAAGGCATCTGAGGACTTAAAGAAGCATGGTGCCTGTGCTCACCGCCCTGGGTGGCATCCTT
AAGAAGAAGGGGCATCATGAGGCAGAGATTAAGCCCCTGGCACAGTCGCATGCCACCAAGCACAAGATCCCCGTGAAGTACCT..3` -2bp deletion= **-2bp** TAA: premature stop codon
Modified Allele 2
5`..CTCTTTAAGGGTCACCCAGAGACTCTGGAGAAGTTTGACAAGTTCAAGCACCTGAAGTCAGAGGACGAGATGAAGNANGGNGCCNCTGNGCTCACCGCCCTGGGTGGCATCCTTAAGAAGAAGGGGCATCA
TGAGGCAGAGATTAANCCCCTGGCACAGTCNCATGCCACCAAGCACAANATCCCNGTGAAGTACCT.. 3` -21bp insertion= **-21bp**

MB protein sequence of SKBR3 MBko2
Modified allele 1
MGLSDGEWQLVLNVWGKVEADIPGHGQEVLIRLFKGHPETLEKFDKFKHLKSEDEMKASEDLKKHGA truncated with lack of distal histidine → **Loss of function**
Modified allele 2
missense mutation, no protein is produced → **Loss of function**

Supplemental Figure S2. Sequencing of MCF7 and SKBR3 MB-knockout cells shows genomic alterations

(**A**) DNA and protein sequences of wildtype human *MB* exon 2, showing induced modification with resulting genetic alteration and loss of function proteins in MCF7 MB-KO cells. (**B**) and (**C**) the same as (**A**) but in SKBR3 MB-KO cells.

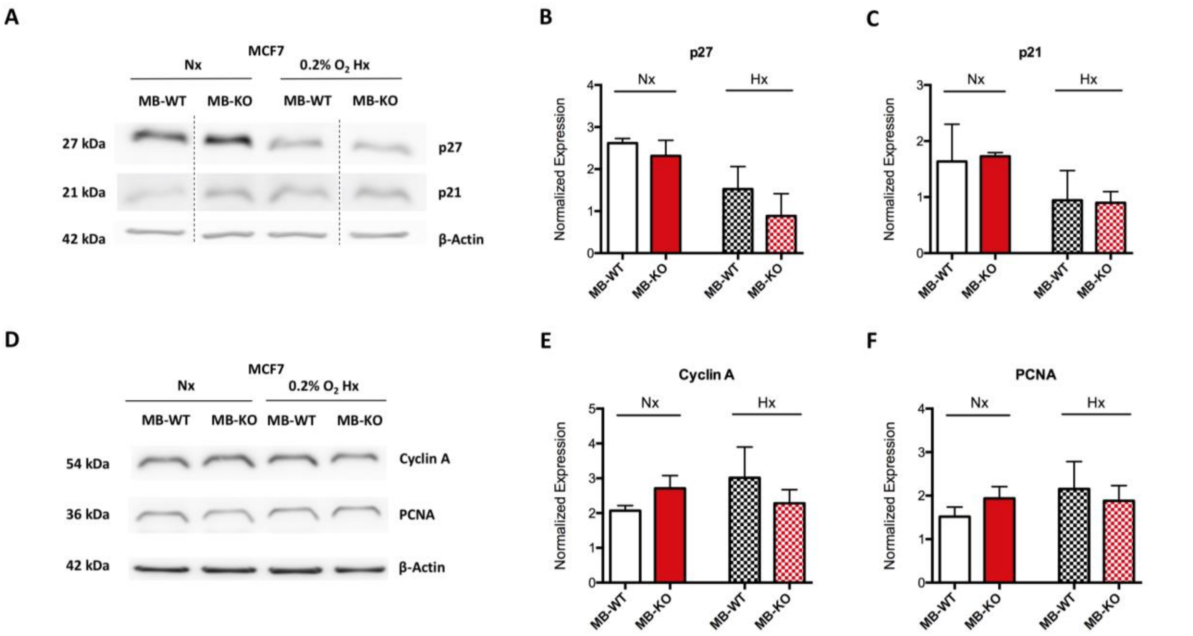

Supplemental Figure S3. Myoglobin does not impact p27, p21, Cyclin A, and PCNA proteins expressions

(**A**) and (**D**) Representative Western Blotting image of whole tissue lysate of MB-WT and MB-KO clones of MCF7 cells cultured at normoxia Nx or 0.2% O_2 Hx for 72h and stained for (**A**) p27 (27 kDa), p21 (21 kDa) and β-actin (42 kDa) used as loading control or (**D**) Cyclin A (54 kDa), PCNA (36kDa) and β-actin (42 kDa) used as the loading control. Panels (**B**), (**C**), (**E**), and (**F**) Band intensity of p27, p21, cyclin A and PCNA proteins, respectively, after Western Blotting, from MB-WT (white) and MB-KO (red) cells of MCF7 at Nx (empty bars) and 0.2%O_2 Hx (dashed bars), was quantified using MCID Analysis 7.0 and normalized to β-actin (n=3). Data are shown as bar graph with mean and standard error of mean and analyzed by Student`s t-test

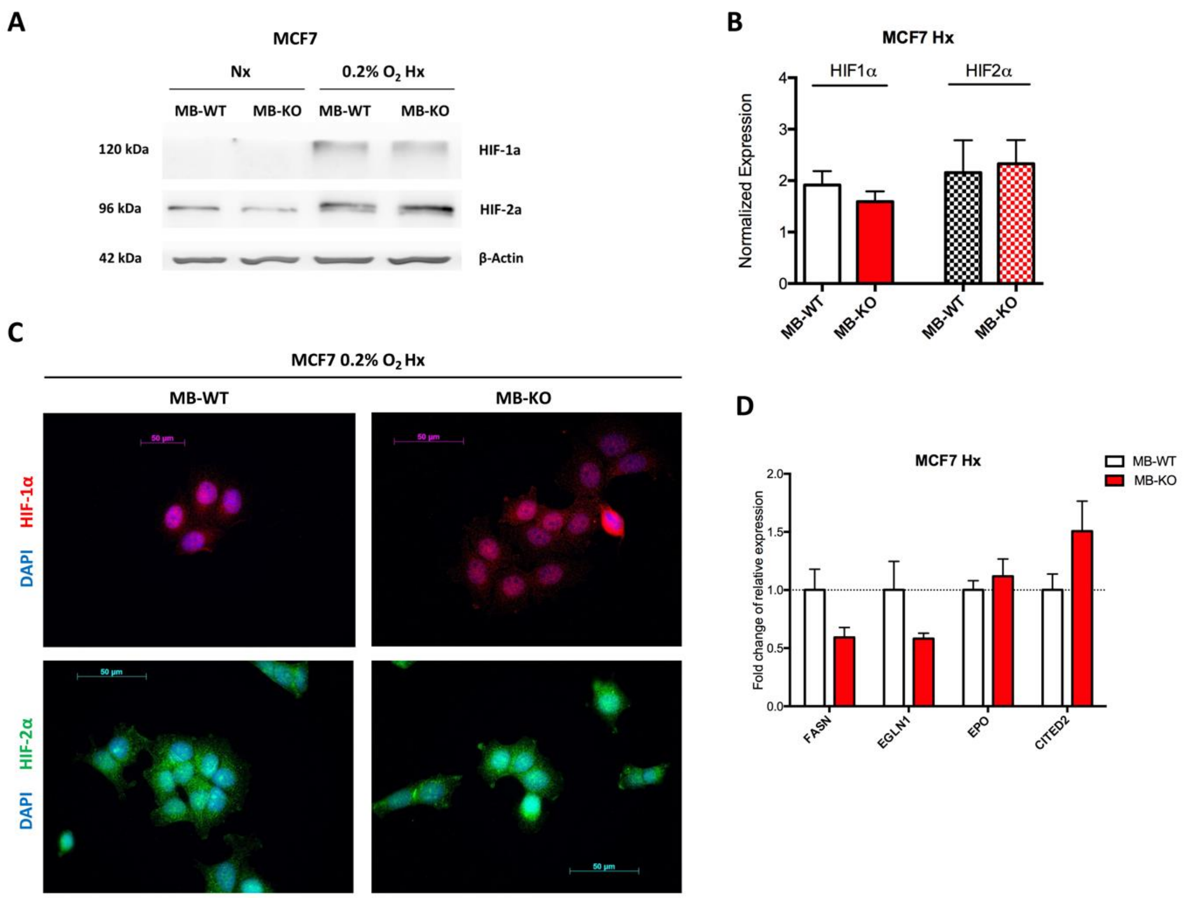

Supplemental Figure S4. Endogenously expressed MB in BrCa cells has no impact on cellular response to hypoxia.

(**A**) Representative Western Blotting image (n=3) of whole tissue lysate of MB-WT and MB-KO clones of MCF7 cells cultured at normoxia Nx or 0.2% O_2 Hx for 72h and stained for hypoxia-inducible factor-1a (HIF-1α) (120 kDa), hypoxia-inducible factor-2a (HIF-2α) (96 kDa) and β-actin (42 kDa) used as the loading control. (**B**) Band intensity of HIF-1α (empty bars) and HIF-2α (dashed bars) proteins after Western Blotting, from MB-WT (white) and MB-KO (red) cells at 0.2%O_2 Hx for 72h, was quantified using MCID Analysis 7.0 and normalized to β-actin (n=3). (**C**) Representative immunocytochemistry images of MB-WT and MB-KO MCF7 cells stained for HIF-1α (red), HIF-2α (green), and DAPI (blue) after culturing at 0.2% O_2 hypoxia for 72h. Scale bar is 50µm. (**D**) Relative mRNA expression levels of downstream target genes of hypoxia-inducible factors: *FASN, EGLN1, EPO, and CITED2*, quantified by qPCR and normalized to β-actin (*ACTB*) mRNA expression levels, from MB-WT and MB-KO clones of MCF7 cells cultured at 0.2% O_2 Hx, for 72h. (n=3 per group). Data are shown as bar graphs and are presented as mean and standard error of mean and analyzed by Student t-test ***$p<0.001$

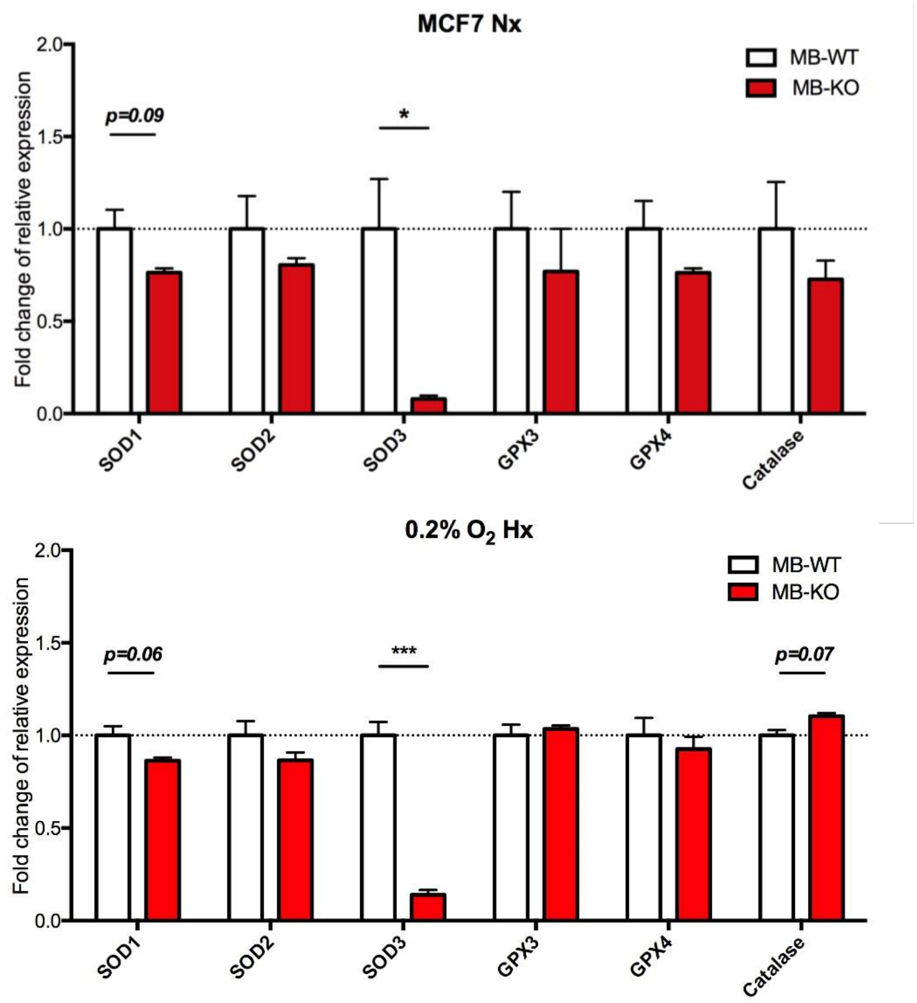

Supplemental Figure S5. Loss of Myoglobin downregulates SOD3 antioxidant gene expression in breast cancer cells

Relative mRNA expression levels of antioxidants genes: superoxide dismutase 1-3 (*SOD1, SOD2, SOD3*) Glutathione peroxidase 3 and 4 (*GPX3, GPX4*), and Catalase, quantified by qPCR and normalized to β-actin (*ACTB*) mRNA expression levels, from MB-WT (white bars) and MB-KO (red bars) clones of MCF7 cells cultured at normoxia (upper panel) and 0.2% O_2 hypoxia (lower panel) for 72h (n=3 per group). Data are shown as bar graphs and are presented as mean and standard error of mean and analyzed by Student's t-test *p<0.05

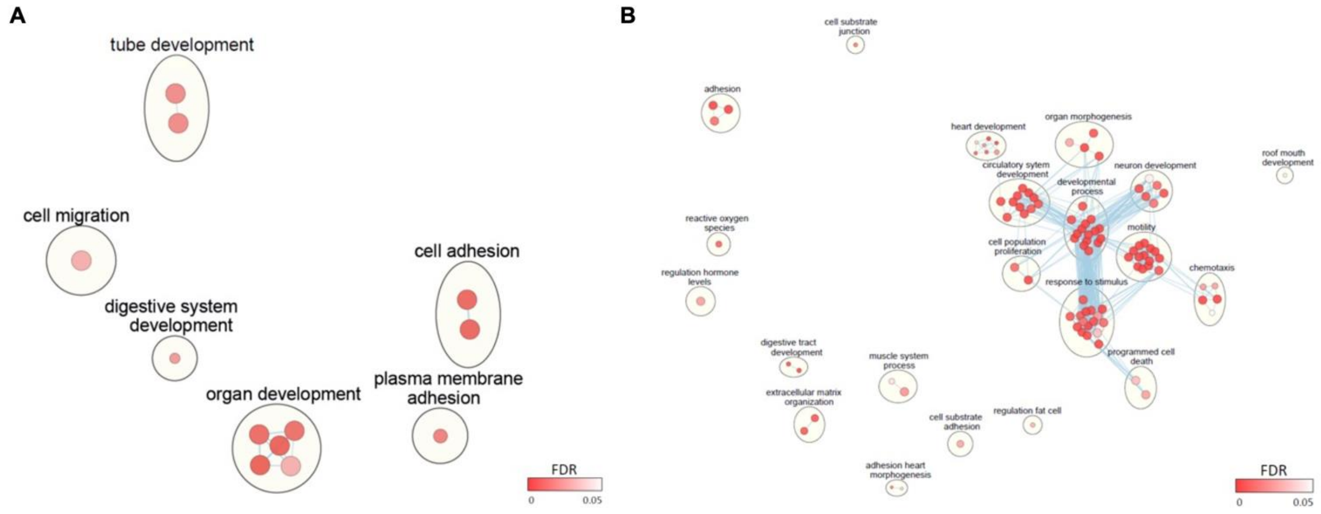

Supplemental Figure S6. Enrichment maps for (A) normoxia and (B) hypoxia datasets

Each node represents a single GO-term. Blue lines are drawn between terms with a Jaccard-Index < 0.25. The FDR is represented by the node color. Annotations were created using AutoAnnotate and adjusted by hand. Clusters were arranged using CoSE algorithm, scaled and adjusted by hand.

Supplementary Tables

Supplementary table S1. List of all altered genes for the normoxia dataset.

Ensembl_ID	GeneName	log2FoldChange	padj
ENSG00000136859	ANGPTL2	6.497238777	8.29E-19
ENSG00000085276	MECOM	1.908155009	1.19E-16
ENSG00000170689	HOXB9	2.370505195	2.13E-14
ENSG00000102271	KLHL4	2.27965335	4.09E-14
ENSG00000186583	SPATC1	-2.367750837	2.13E-11
ENSG00000003436	TFPI	1.877245487	2.13E-11
ENSG00000254535	PABPC4L	2.067369791	2.13E-11
ENSG00000047634	SCML1	2.108920653	2.13E-11
ENSG00000204791	SMPD5	-2.450028613	3.42E-11
ENSG00000147256	ARHGAP36	2.010617272	1.84E-09
ENSG00000111799	COL12A1	-1.514412518	8.15E-09
ENSG00000254337	-	1.186863904	2.37E-08
ENSG00000170509	HSD17B13	3.383238673	5.00E-08
ENSG00000286322	-	7.950572644	2.33E-07
ENSG00000069020	MAST4	1.166467697	2.93E-07
ENSG00000116729	WLS	2.472628126	8.19E-07
ENSG00000099204	ABLIM1	1.272415642	1.07E-06
ENSG00000100867	DHRS2	-1.348192716	1.15E-06

ENSG00000205426	KRT81	-1.605372399	1.33E-06
ENSG00000156463	SH3RF2	1.30697382	1.58E-06
ENSG00000115221	ITGB6	1.179023437	2.10E-06
ENSG00000165025	SYK	0.816603319	2.19E-06
ENSG00000177283	FZD8	1.224943999	2.68E-06
ENSG00000101986	ABCD1	-1.097831462	3.11E-06
ENSG00000006747	SCIN	1.468030029	3.72E-06
ENSG00000204442	FAM155A	1.436506374	5.09E-06
ENSG00000142609	CFAP74	1.169826068	5.59E-06
ENSG00000164125	GASK1B	1.202933479	5.96E-06
ENSG00000164749	HNF4G	2.461734879	5.96E-06
ENSG00000169851	PCDH7	-1.282026173	6.53E-06
ENSG00000138386	NAB1	1.279430505	8.01E-06
ENSG00000164649	CDCA7L	0.852166284	9.64E-06
ENSG00000078053	AMPH	1.130350158	1.22E-05
ENSG00000112246	SIM1	2.237232903	2.13E-05
ENSG00000230221	-	5.245912348	2.38E-05
ENSG00000090661	CERS4	-0.966670875	2.75E-05
ENSG00000106688	SLC1A1	-1.096463336	4.56E-05
ENSG00000164690	SHH	-0.783392357	4.56E-05
ENSG00000084444	FAM234B	-0.82863806	4.65E-05
ENSG00000168542	COL3A1	1.785512546	4.65E-05
ENSG00000196139	AKR1C3	1.277371363	4.97E-05
ENSG00000140465	CYP1A1	-1.799779936	5.61E-05
ENSG00000216740	ANXA2P3	4.807368885	8.17E-05
ENSG00000235524	MTCO1P23	5.734933587	9.14E-05
ENSG00000169403	PTAFR	-1.204234084	9.25E-05
ENSG00000172575	RASGRP1	0.896350457	0.000121639
ENSG00000144366	GULP1	1.219525523	0.000133636
ENSG00000242100	RPL9P32	4.008219705	0.000148665
ENSG00000105388	CEACAM5	1.310571185	0.000177306
ENSG00000249264	EEF1A1P9	2.398701091	0.000177306
ENSG00000219747	RPL32P16	3.985066689	0.000192152
ENSG00000244192	-	4.016477241	0.000207848
ENSG00000019549	SNAI2	1.914653463	0.000230149
ENSG00000174473	GALNTL6	-1.056506923	0.000230266
ENSG00000135111	TBX3	1.579923431	0.000246576
ENSG00000242353	RPL12P30	4.488389205	0.000303016
ENSG00000177728	TMEM94	-1.172667797	0.000339375
ENSG00000260290	-	2.938784122	0.000339375
ENSG00000177707	NECTIN3	1.088425619	0.000386683
ENSG00000145819	ARHGAP26	0.806239319	0.000438049
ENSG00000218426	-	3.952504531	0.000438049
ENSG00000236570	RAD23BP1	4.668381483	0.000438049
ENSG00000146674	IGFBP3	1.103187633	0.000456235

ENSG00000224897	POT1-AS1	1.32339549	0.000471726
ENSG00000110934	BIN2	2.175411642	0.000486887
ENSG00000198189	HSD17B11	1.364153758	0.000529167
ENSG00000235962	RPL7AP53	3.518718481	0.000533354
ENSG00000250241	-	1.091674409	0.000560154
ENSG00000099994	SUSD2	-1.083682308	0.000605718
ENSG00000196932	TMEM26	1.514357332	0.000605718
ENSG00000221923	ZNF880	1.313214424	0.000628899
ENSG00000152661	GJA1	-1.396003408	0.000684844
ENSG00000115590	IL1R2	2.302150766	0.000684844
ENSG00000211689	TRGC1	-1.589460016	0.000719756
ENSG00000198363	ASPH	0.990306265	0.00073226
ENSG00000085831	TTC39A	-0.643551654	0.000735714
ENSG00000130700	GATA5	3.662812649	0.000735714
ENSG00000138650	PCDH10	0.707766674	0.000760099
ENSG00000130827	PLXNA3	-0.880510779	0.000760418
ENSG00000253506	NACA2	3.230536532	0.000775503
ENSG00000139793	MBNL2	0.791411677	0.00078536
ENSG00000218283	MORF4L1P1	2.186681861	0.00078536
ENSG00000226396	-	2.586801472	0.00078536
ENSG00000147689	FAM83A	-1.052500264	0.00079754
ENSG00000182575	NXPH3	-0.881844501	0.00079754
ENSG00000250148	KRT8P31	3.137297838	0.00079754
ENSG00000167925	GHDC	-1.122806256	0.000806164
ENSG00000152137	HSPB8	-0.919015119	0.000843577
ENSG00000183049	CAMK1D	0.869017519	0.000862106
ENSG00000105971	CAV2	1.455190949	0.000876284
ENSG00000144824	PHLDB2	1.356770507	0.000881501
ENSG00000189212	DPY19L2P1	1.863555495	0.000890374
ENSG00000223803	RPS20P14	4.192687179	0.000922288
ENSG00000188460	ACTBP11	3.113010507	0.00105501
ENSG00000250859	HNRNPKP1	2.552120777	0.001265679
ENSG00000143341	HMCN1	0.928470086	0.001314871
ENSG00000228499	TMSB10P1	4.757400682	0.001345375
ENSG00000180764	PIPSL	2.729762359	0.001390165
ENSG00000233668	-	3.289256155	0.001390165
ENSG00000179862	CITED4	-1.256261148	0.001407471
ENSG00000204253	HNRNPCP2	3.046251339	0.001407471
ENSG00000108018	SORCS1	-1.481608293	0.001437959
ENSG00000115641	FHL2	-0.796786116	0.001437959
ENSG00000196083	IL1RAP	0.841637095	0.001452918
ENSG00000232385	RPS3AP25	5.005671527	0.001469175
ENSG00000196344	ADH7	3.961766052	0.001478951
ENSG00000074855	ANO8	-0.65265462	0.001525634
ENSG00000231767	RPS27AP5	3.609865418	0.00156887

ENSG00000170786	SDR16C5	1.344517441	0.001711233
ENSG00000183044	ABAT	-0.485585083	0.0017634
ENSG00000249092	PPIAP77	4.691641365	0.001769923
ENSG00000134668	SPOCD1	-1.359573419	0.001814703
ENSG00000122644	ARL4A	1.123161768	0.001814703
ENSG00000178715	-	3.325417444	0.00182207
ENSG00000135549	PKIB	-0.693523525	0.001835685
ENSG00000143878	RHOB	-0.650569354	0.001977912
ENSG00000146648	EGFR	0.76305769	0.00198893
ENSG00000112655	PTK7	-0.632518228	0.002021276
ENSG00000160867	FGFR4	-0.827727153	0.00204014
ENSG00000184809	B3GALT5-AS1	-1.137284769	0.002097184
ENSG00000121552	CSTA	-1.075593353	0.002172879
ENSG00000237433	RPSAP11	4.753033069	0.002174838
ENSG00000145632	PLK2	-0.501831328	0.002209346
ENSG00000228981	-	3.402845934	0.002209346
ENSG00000233830	EIF4HP1	3.583713708	0.002296601
ENSG00000185973	TMLHE	-0.647402841	0.002425823
ENSG00000092758	COL9A3	-1.65140853	0.002511586
ENSG00000135333	EPHA7	0.945804706	0.002511586
ENSG00000223873	SAP18P2	3.909606545	0.002511586
ENSG00000147255	IGSF1	1.296410742	0.002537293
ENSG00000146757	ZNF92	0.92664326	0.002765914
ENSG00000069667	RORA	1.382603025	0.002958463
ENSG00000247763	TUBAP14	3.251048421	0.002958463
ENSG00000239622	-	3.598251135	0.002958463
ENSG00000240051	RPL23AP10	5.27111787	0.002958463
ENSG00000095321	CRAT	-0.779497345	0.002967373
ENSG00000277443	MARCKS	-0.591580403	0.00308335
ENSG00000248415	GAPDHP61	2.704600268	0.003105153
ENSG00000214193	SH3D21	-0.5724357	0.00314674
ENSG00000111058	ACSS3	0.912031399	0.003302889
ENSG00000249855	EEF1A1P19	2.068143946	0.003302889
ENSG00000111052	LIN7A	1.18096491	0.003378816
ENSG00000230629	RPS23P8	2.045316003	0.003993038
ENSG00000013619	MAMLD1	-0.965448116	0.004011967
ENSG00000102024	PLS3	2.381712125	0.004011967
ENSG00000179083	FAM133A	-1.245266069	0.004079696
ENSG00000228929	RPS13P2	3.705910164	0.004090232
ENSG00000240882	-	4.42688634	0.004727095
ENSG00000245205	EEF1A1P4	2.042503359	0.004751799
ENSG00000283041	-	1.424514358	0.004901767
ENSG00000230783	RPS3AP13	3.658027714	0.004928121
ENSG00000168843	FSTL5	-1.158745389	0.005002829
ENSG00000254038	-	1.554115143	0.005116486

ENSG00000164850	GPER1	-0.959181296	0.005178702
ENSG00000232346	-	3.551134423	0.005178702
ENSG00000116774	OLFML3	-1.100370809	0.005186402
ENSG00000228834	ATP5MFP2	4.925243198	0.005196716
ENSG00000196636	SDHAF3	-1.514943742	0.005303141
ENSG00000254241	MTCO1P47	4.498863591	0.005303141
ENSG00000255450	-	3.368729641	0.005451173
ENSG00000235065	RPL24P2	2.207403318	0.005484245
ENSG00000233057	EEF1A1P14	2.382147084	0.005599921
ENSG00000213293	-	3.777378219	0.005599921
ENSG00000099954	CECR2	1.329473206	0.005992344
ENSG00000117525	F3	1.470959893	0.005992344
ENSG00000234782	TPT1P9	1.940233589	0.00637564
ENSG00000237709	EEF1A1P28	2.592000439	0.006622458
ENSG00000139239	RPL14P1	2.163965933	0.006667098
ENSG00000219932	RPL12P8	4.579332181	0.006851717
ENSG00000143995	MEIS1	0.803993311	0.007115745
ENSG00000217889	KRT18P48	2.746072725	0.007340391
ENSG00000118922	KLF12	0.813612547	0.007397212
ENSG00000128928	IVD	-0.502307335	0.007399525
ENSG00000137193	PIM1	0.698145337	0.007399525
ENSG00000183346	CABCOCO1	2.05425382	0.007399525
ENSG00000240121	RPS27P20	5.933269813	0.007399525
ENSG00000154553	PDLIM3	1.694268806	0.007428032
ENSG00000103485	QPRT	-1.678353536	0.007468798
ENSG00000215835	-	3.324637792	0.007909283
ENSG00000128422	KRT17	-2.224527163	0.007920521
ENSG00000185607	ACTBP7	3.136074151	0.008004505
ENSG00000111339	ART4	1.569154834	0.008200124
ENSG00000240522	RPL7AP10	2.067016399	0.008433607
ENSG00000198753	PLXNB3	-1.041520769	0.008439501
ENSG00000227578	RPS3AP53	5.565143512	0.008439501
ENSG00000058091	CDK14	0.936951985	0.008668802
ENSG00000226543	MYL6P1	5.863341966	0.008838427
ENSG00000119699	TGFB3	-0.678634827	0.008871155
ENSG00000226243	RPL37AP1	2.78872679	0.008883673
ENSG00000101188	NTSR1	-0.842785383	0.009242164
ENSG00000213750	-	4.868422039	0.009242164
ENSG00000266602	-	-1.7324115	0.009329989
ENSG00000099889	ARVCF	-0.696148033	0.009329989
ENSG00000240622	RPL7P15	3.249642894	0.009329989
ENSG00000170545	SMAGP	0.827212584	0.009375689
ENSG00000219507	FTH1P8	1.774593631	0.009394364
ENSG00000205978	NYNRIN	-0.959789906	0.009413676
ENSG00000253341	PCBP2P2	2.186444986	0.009413676

ENSG00000203971	-	5.776230155	0.009413676
ENSG00000099256	PRTFDC1	0.795507266	0.009494976
ENSG00000216365	RPL37P15	4.920851347	0.009520006
ENSG00000238116	RAD21P1	3.831686229	0.010033925
ENSG00000127585	FBXL16	-0.729623046	0.010242732
ENSG00000179918	SEPHS2	-0.512902904	0.010377263
ENSG00000107518	ATRNL1	0.994050619	0.010659626
ENSG00000227121	LINC02672	1.615020976	0.011095342
ENSG00000240828	RPL21P4	5.095907035	0.011485106
ENSG00000259032	ENSAP2	3.100431495	0.011713151
ENSG00000215218	UBE2QL1	-1.025684813	0.012520138
ENSG00000204287	HLA-DRA	-1.323224749	0.013111032
ENSG00000187017	ESPN	-0.675830086	0.013111032
ENSG00000111348	ARHGDIB	1.165789744	0.013111032
ENSG00000169253	-	4.020029354	0.013111032
ENSG00000063601	MTMR1	-0.470223816	0.01331066
ENSG00000168269	FOXI1	-1.418331634	0.013537674
ENSG00000164292	RHOBTB3	0.782931885	0.013644114
ENSG00000147955	SIGMAR1	-0.916056083	0.014080568
ENSG00000254416	LINC02732	-0.935101571	0.015055435
ENSG00000173846	PLK3	-0.838196617	0.015241081
ENSG00000241258	CRCP	0.423543218	0.015241081
ENSG00000076356	PLXNA2	0.964900772	0.015241081
ENSG00000139211	AMIGO2	1.043845458	0.015346655
ENSG00000107957	SH3PXD2A	0.69644889	0.015574357
ENSG00000071859	FAM50A	-0.63153829	0.01642141
ENSG00000104549	SQLE	-0.427854588	0.01642141
ENSG00000187122	SLIT1	-1.215226808	0.016833947
ENSG00000197008	ZNF138	0.597633962	0.017120482
ENSG00000228818	-	2.682027264	0.017120482
ENSG00000106609	TMEM248	0.370311854	0.017159749
ENSG00000228247	UBBP2	2.957209864	0.017533719
ENSG00000242607	RPS3AP34	6.097316364	0.017622727
ENSG00000173852	DPY19L1	0.678400585	0.017636161
ENSG00000196437	ZNF569	-1.731047377	0.017639448
ENSG00000228462	RPS19P7	4.15142014	0.017639448
ENSG00000100346	CACNA1I	-0.939490968	0.017905363
ENSG00000049089	COL9A2	-0.716433064	0.017905363
ENSG00000236946	HNRNPA1P70	4.220827587	0.017905363
ENSG00000269516	CYP4F23P	-1.355200061	0.018176628
ENSG00000228232	GAPDHP1	1.926244389	0.018249316
ENSG00000234067	RPL5P10	5.256489928	0.018249316
ENSG00000157303	SUSD3	-0.785217633	0.01826698
ENSG00000224899	LINC02830	1.378909107	0.018290874
ENSG00000127954	STEAP4	-1.10247217	0.018468408

ENSG00000224411	HSP90AA2P	2.835160673	0.018468408
ENSG00000215388	ACTG1P3	3.173071442	0.018468408
ENSG00000102312	PORCN	-0.650335557	0.018669646
ENSG00000183778	B3GALT5	-0.983935411	0.018819701
ENSG00000254270	ERHP1	4.157242204	0.018819701
ENSG00000259078	PTBP1P	5.687193611	0.018819701
ENSG00000197121	PGAP1	1.001153218	0.018853073
ENSG00000103197	TSC2	-0.522476006	0.01919956
ENSG00000213790	OLA1P1	2.498760921	0.01919956
ENSG00000102125	TAZ	-0.66520731	0.019442471
ENSG00000197774	EME2	-0.614885155	0.019442471
ENSG00000184271	POU6F1	1.17600574	0.019442471
ENSG00000214460	TPT1P6	2.410441562	0.019442471
ENSG00000226549	SCDP1	3.315406452	0.019442471
ENSG00000227615	-	3.403343099	0.019442471
ENSG00000280195	-	-0.765248941	0.019539965
ENSG00000227968	BUB3P1	4.316965811	0.020335929
ENSG00000127955	GNAI1	-1.059385072	0.020356509
ENSG00000270706	PRMT1P1	3.454672059	0.020449478
ENSG00000231181	-	2.984186863	0.020823463
ENSG00000240535	-	4.887889583	0.020823463
ENSG00000198406	BZW1P2	2.054100395	0.021161179
ENSG00000242411	-	2.791384264	0.021165677
ENSG00000071553	ATP6AP1	-0.615086467	0.021198717
ENSG00000230807	-	4.185157487	0.021198717
ENSG00000114450	GNB4	1.449291301	0.021323853
ENSG00000236480	PKMP1	2.497019359	0.021323853
ENSG00000230916	MTCO1P53	1.366620002	0.02175371
ENSG00000242327	-	2.885314351	0.021781039
ENSG00000277043	EEF1A1P42	3.046001874	0.021865622
ENSG00000234332	BCAS2P2	3.019850599	0.021904441
ENSG00000152217	SETBP1	1.292835226	0.022309564
ENSG00000228887	EEF1DP1	2.1392636	0.022392867
ENSG00000259706	HSP90B2P	1.863842508	0.022407127
ENSG00000113594	LIFR	0.949861413	0.022720959
ENSG00000005001	PRSS22	-0.718451213	0.023054112
ENSG00000214078	CPNE1	-0.625904009	0.023204676
ENSG00000113580	NR3C1	0.733982059	0.023717783
ENSG00000225356	-	2.570596956	0.023792208
ENSG00000183665	TRMT12	-0.446000179	0.023802567
ENSG00000164236	ANKRD33B	1.295901116	0.024034587
ENSG00000213891	RPL3P6	2.910977628	0.024034587
ENSG00000223668	EEF1A1P24	1.390348206	0.02405663
ENSG00000232054	NPM1P34	4.027888676	0.024209386
ENSG00000149573	MPZL2	0.624298682	0.024327882

ENSG00000163513	TGFBR2	0.687717999	0.025182507
ENSG00000172974	VDAC2P5	3.040203602	0.02557041
ENSG00000059122	FLYWCH1	-0.535597772	0.026359973
ENSG00000132329	RAMP1	-1.151709045	0.026496047
ENSG00000124762	CDKN1A	-0.816297241	0.026867409
ENSG00000230146	SEPHS1P4	2.824122165	0.027318018
ENSG00000162004	CCDC78	-0.609386531	0.027888671
ENSG00000244503	-	2.927566729	0.027888671
ENSG00000213613	RPL11P3	2.073794128	0.027954729
ENSG00000162066	AMDHD2	-0.746617285	0.028088891
ENSG00000263266	RPS7P1	3.304406215	0.028088891
ENSG00000146700	SSC4D	-1.077006632	0.028297426
ENSG00000165905	LARGE2	-0.70083752	0.028297426
ENSG00000196715	VKORC1L1	0.523888854	0.028297426
ENSG00000223529	EEF1A1P8	1.565219032	0.028297426
ENSG00000223739	RPS15AP15	2.48829157	0.028297426
ENSG00000248373	-	-1.915693033	0.028361297
ENSG00000145451	GLRA3	0.953610434	0.028377457
ENSG00000165895	ARHGAP42	0.763993188	0.028740261
ENSG00000234882	EIF3EP1	1.805475621	0.02932064
ENSG00000187957	DNER	-1.689575438	0.029559212
ENSG00000218582	GAPDHP63	2.333091969	0.029736285
ENSG00000233111	RAB1C	2.929796171	0.029736285
ENSG00000224773	HSPA8P7	3.521345274	0.029736285
ENSG00000250363	KRT18P21	2.892056218	0.029934936
ENSG00000233476	EEF1A1P6	2.372856072	0.030843593
ENSG00000225568	-	3.690815517	0.031658775
ENSG00000283057	-	4.528258581	0.031999332
ENSG00000100842	EFS	-1.330019831	0.032803633
ENSG00000171798	KNDC1	-0.573539678	0.03305499
ENSG00000213704	EEF1A1P15	2.384751765	0.034213815
ENSG00000227051	C14orf132	1.229861066	0.034315347
ENSG00000225536	STIP1P3	3.815814155	0.034629087
ENSG00000117859	OSBPL9	-0.437030595	0.035140735
ENSG00000250144	-	1.898506926	0.035140735
ENSG00000262152	LINC00514	-0.824478928	0.035352576
ENSG00000218175	-	2.440967361	0.035352576
ENSG00000230391	RPSAP23	3.711109477	0.035352576
ENSG00000234589	-	3.606304299	0.035479072
ENSG00000146072	TNFRSF21	0.424860354	0.035623349
ENSG00000179715	PCED1B	1.022584521	0.035623349
ENSG00000101276	SLC52A3	-0.682977192	0.036331725
ENSG00000069974	RAB27A	0.763178171	0.036331725
ENSG00000231494	RPL21P35	4.719297597	0.036435016
ENSG00000170153	RNF150	0.776678978	0.037092688

ENSG00000135766	EGLN1	-0.601030685	0.037286484
ENSG00000257616	-	3.292847871	0.037286484
ENSG00000064787	BCAS1	1.968671816	0.037702376
ENSG00000267398	-	4.86359512	0.037870512
ENSG00000197044	ZNF441	-2.33601766	0.037981597
ENSG00000123472	ATPAF1	-0.355171675	0.037981597
ENSG00000151846	PABPC3	2.307863355	0.037981597
ENSG00000185825	BCAP31	-0.597966213	0.038173178
ENSG00000179698	WDR97	-0.734068206	0.038768233
ENSG00000242683	RPL12P21	4.259733953	0.039259136
ENSG00000115129	TP53I3	-0.781413595	0.039368471
ENSG00000137573	SULF1	0.773326094	0.039368471
ENSG00000223810	KRT8P28	5.037443263	0.039391096
ENSG00000184226	PCDH9	0.520495288	0.039856514
ENSG00000240480	RPL29P2	2.753668271	0.04048991
ENSG00000132746	ALDH3B2	-0.774910521	0.040972325
ENSG00000167962	ZNF598	-0.482599097	0.041518364
ENSG00000197043	ANXA6	-0.733185227	0.041586235
ENSG00000213820	RPL13P2	2.88606589	0.041586235
ENSG00000004948	CALCR	1.150273569	0.041707306
ENSG00000242291	RPL36AP51	2.544627727	0.041707306
ENSG00000227309	-	3.213063253	0.041707306
ENSG00000251333	RTN3P1	3.435185086	0.041707306
ENSG00000243064	ABCC13	5.059031564	0.041707306
ENSG00000167768	KRT1	2.738495237	0.041949884
ENSG00000216713	MTND4P13	5.327619491	0.041949884
ENSG00000100003	SEC14L2	-0.800313034	0.042679684
ENSG00000167996	FTH1	0.665237636	0.042785064
ENSG00000178028	DMAP1	-0.373834026	0.043141034
ENSG00000196814	MVB12B	0.665158879	0.043141034
ENSG00000242299	-	1.646650805	0.043141034
ENSG00000224333	GAPDHP20	3.544311814	0.043141034
ENSG00000270553	-	4.02133657	0.043141034
ENSG00000220472	-	3.019992559	0.043271716
ENSG00000265480	KRT18P55	3.122907463	0.043271716
ENSG00000243547	HNRNPKP4	1.841565268	0.043302499
ENSG00000213601	KRT18P19	2.467937009	0.04362776
ENSG00000151692	RNF144A	0.817176493	0.04378853
ENSG00000117385	P3H1	-0.674163707	0.043794529
ENSG00000168140	VASN	-0.893799166	0.043943537
ENSG00000234785	EEF1GP5	2.010451742	0.044073584
ENSG00000260711	-	1.022344849	0.044077435
ENSG00000225971	RPS3AP51	3.451834984	0.044077435
ENSG00000102683	SGCG	-0.941271962	0.044318398
ENSG00000092929	UNC13D	-0.552547603	0.045166763

ENSG00000132561	MATN2	-0.459894097	0.046008225
ENSG00000236686	BZW1P1	3.134549276	0.046008225
ENSG00000232042	-	4.68501998	0.046226247
ENSG00000232493	RPL12P11	2.716425319	0.046356949
ENSG00000167967	E4F1	-0.505564179	0.046711014
ENSG00000138821	SLC39A8	-1.005540128	0.04717101
ENSG00000154274	C4orf19	0.537463886	0.04726818
ENSG00000145824	CXCL14	-2.432576793	0.047492552
ENSG00000108679	LGALS3BP	-0.575857397	0.047492552
ENSG00000008256	CYTH3	0.401236013	0.047492552
ENSG00000232883	-	2.077703722	0.047492552
ENSG00000261557	EEF1A1P38	1.817046237	0.047551752
ENSG00000184100	BRD7P2	4.445268476	0.047685988
ENSG00000089820	ARHGAP4	-0.590067909	0.047949638
ENSG00000160293	VAV2	-0.392153137	0.047949638
ENSG00000226581	LINC02848	2.313287097	0.047949638
ENSG00000255642	PABPC1P4	1.8582801	0.048064447
ENSG00000258162	-	2.992999721	0.048532021
ENSG00000165731	RET	-0.656388536	0.048731744
ENSG00000236937	PTGES3P4	3.698104157	0.048731744
ENSG00000268282	-	3.090519817	0.048913995
ENSG00000151572	ANO4	4.560214531	0.048937848
ENSG00000254387	MYL12AP1	4.626925986	0.04906469
ENSG00000181085	MAPK15	-0.682094944	0.049123345
ENSG00000173917	HOXB2	0.954410081	0.049346147
ENSG00000141527	CARD14	-0.521667592	0.049377932
ENSG00000163017	ACTG2	-1.044928379	0.049958593
ENSG00000090674	MCOLN1	-0.602387236	0.049958593
ENSG00000115594	IL1R1	0.895353232	0.049958593
ENSG00000232187	FTH1P7	1.874467012	0.049958593
ENSG00000243094	RPL32P2	2.443087852	0.049958593

Supplementary table S2. List of all altered genes for the hypoxia dataset.

Ensembl_ID	GeneName	log2FoldChange	padj
ENSG00000115221	ITGB6	1.887537323	1.17E-22
ENSG00000003436	TFPI	2.172972248	6.06E-17
ENSG00000146674	IGFBP3	1.773300934	5.30E-13
ENSG00000165025	SYK	1.163683373	5.66E-13
ENSG00000154229	PRKCA	1.163129691	2.21E-10
ENSG00000164683	HEY1	1.481845222	6.36E-10
ENSG00000172575	RASGRP1	1.265181902	8.15E-10
ENSG00000169851	PCDH7	-1.647079526	8.38E-10
ENSG00000085276	MECOM	1.51082496	2.20E-08

ENSG00000136859	ANGPTL2	8.129998244	3.77E-08
ENSG00000164749	HNF4G	2.636267727	3.91E-08
ENSG00000196083	IL1RAP	1.197383809	5.39E-08
ENSG00000142609	CFAP74	1.399515499	5.67E-08
ENSG00000146072	TNFRSF21	0.774901922	7.61E-08
ENSG00000116729	WLS	2.827810124	7.61E-08
ENSG00000154783	FGD5	1.965559587	1.06E-07
ENSG00000111799	COL12A1	-1.540203088	1.13E-07
ENSG00000186583	SPATC1	-1.931076585	1.41E-07
ENSG00000047634	SCML1	1.65194411	1.28E-06
ENSG00000146648	EGFR	0.948714015	1.55E-06
ENSG00000144824	PHLDB2	1.727146526	1.61E-06
ENSG00000164292	RHOBTB3	1.207442733	4.87E-06
ENSG00000198918	RPL39	1.450868842	4.87E-06
ENSG00000012779	ALOX5	1.597723393	1.13E-05
ENSG00000058085	LAMC2	0.806616786	1.14E-05
ENSG00000121552	CSTA	-1.416173976	1.34E-05
ENSG00000006747	SCIN	1.420886012	1.34E-05
ENSG00000231298	MANCR	2.51434657	1.80E-05
ENSG00000183696	UPP1	1.58692632	2.45E-05
ENSG00000182795	C1orf116	1.600709084	4.38E-05
ENSG00000115919	KYNU	-1.042762853	4.89E-05
ENSG00000107159	CA9	-1.421993728	4.93E-05
ENSG00000169071	ROR2	1.417465636	4.93E-05
ENSG00000135111	TBX3	1.598241824	4.93E-05
ENSG00000064042	LIMCH1	1.267690171	5.04E-05
ENSG00000152137	HSPB8	-1.134438072	5.14E-05
ENSG00000178726	THBD	1.626756766	5.14E-05
ENSG00000107263	RAPGEF1	0.671545037	5.76E-05
ENSG00000285969	-	2.097187313	5.76E-05
ENSG00000286322	-	6.518467235	6.50E-05
ENSG00000163513	TGFBR2	1.007679091	6.65E-05
ENSG00000140465	CYP1A1	-1.56107289	9.77E-05
ENSG00000076641	PAG1	1.563945474	0.000111832
ENSG00000235123	DSCAM-AS1	-0.790080967	0.000113593
ENSG00000057657	PRDM1	2.329038489	0.000145353
ENSG00000204054	LINC00963	0.783798549	0.000179915
ENSG00000204791	SMPD5	-1.633123343	0.000188298
ENSG00000111052	LIN7A	1.33683013	0.000188298
ENSG00000170689	HOXB9	1.905768603	0.000197864
ENSG00000124225	PMEPA1	0.850369061	0.000206504
ENSG00000205426	KRT81	-1.316479878	0.00021194
ENSG00000046604	DSG2	0.787711198	0.000225622
ENSG00000113532	ST8SIA4	-1.050562365	0.000264609
ENSG00000074527	NTN4	1.006042339	0.000264609

ENSG00000080031	PTPRH	1.042134877	0.000281654
ENSG00000266602	-	-2.018787766	0.00028389
ENSG00000168843	FSTL5	-1.800256286	0.000357038
ENSG00000019549	SNAI2	1.698272988	0.000362594
ENSG00000187122	SLIT1	-1.620709612	0.000375597
ENSG00000167925	GHDC	-1.333917599	0.000395162
ENSG00000138386	NAB1	1.082406341	0.000442487
ENSG00000101188	NTSR1	-1.042061506	0.000550814
ENSG00000128052	KDR	1.534894225	0.000623533
ENSG00000138640	FAM13A	1.182037366	0.000662563
ENSG00000198363	ASPH	0.979805823	0.0007227
ENSG00000108018	SORCS1	-1.489535649	0.00077885
ENSG00000147010	SH3KBP1	0.572321065	0.00077885
ENSG00000091136	LAMB1	1.191092795	0.000899757
ENSG00000254038	-	3.003212661	0.001010642
ENSG00000173930	SLCO4C1	-1.288079259	0.001082896
ENSG00000221923	ZNF880	1.335263788	0.001082896
ENSG00000168542	COL3A1	1.602811449	0.001679115
ENSG00000106031	HOXA13	1.016363848	0.001943552
ENSG00000137193	PIM1	0.769454184	0.001981872
ENSG00000053747	LAMA3	0.907185051	0.001981872
ENSG00000105559	PLEKHA4	-1.466672125	0.00198545
ENSG00000116285	ERRFI1	0.685058114	0.002008162
ENSG00000205413	SAMD9	1.418108679	0.002008162
ENSG00000080200	CRYBG3	1.682785382	0.002071108
ENSG00000249267	LINC00939	-3.075744737	0.002190921
ENSG00000166949	SMAD3	0.584936666	0.002190921
ENSG00000145819	ARHGAP26	0.739434387	0.002190921
ENSG00000106609	TMEM248	0.440441705	0.002273963
ENSG00000113594	LIFR	1.206832965	0.002273963
ENSG00000167767	KRT80	0.649454647	0.002723742
ENSG00000156103	MMP16	-1.525939945	0.002925458
ENSG00000176903	PNMA1	0.501869333	0.002925458
ENSG00000258676	-	1.54566665	0.002925458
ENSG00000106258	CYP3A5	1.55131228	0.002925458
ENSG00000260604	-	1.204819417	0.003056491
ENSG00000224897	POT1-AS1	1.22132436	0.003094074
ENSG00000127329	PTPRB	1.921897774	0.003094074
ENSG00000130700	GATA5	5.496109822	0.003113568
ENSG00000162004	CCDC78	-0.773688636	0.003140284
ENSG00000115935	WIPF1	1.458618106	0.003261989
ENSG00000168280	KIF5C	-1.028103024	0.003296233
ENSG00000140479	PCSK6	0.669343311	0.00333866
ENSG00000086548	CEACAM6	1.109644795	0.003627968
ENSG00000145623	OSMR	0.716953336	0.0040397

ENSG00000197261	C6orf141	-1.012853368	0.004144454
ENSG00000156535	CD109	0.848080826	0.004144454
ENSG00000118523	CCN2	1.482978194	0.004161113
ENSG00000112655	PTK7	-0.629473806	0.00420601
ENSG00000100346	CACNA1I	-0.963970906	0.004270001
ENSG00000151632	AKR1C2	-1.016893989	0.004483458
ENSG00000150961	SEC24D	0.545709369	0.005477395
ENSG00000227121	LINC02672	0.909713024	0.005531037
ENSG00000164120	HPGD	1.771775904	0.005531037
ENSG00000171827	ZNF570	-1.077090746	0.005565237
ENSG00000120708	TGFBI	0.695960814	0.005565237
ENSG00000198189	HSD17B11	1.246374525	0.005565237
ENSG00000145779	TNFAIP8	0.746490847	0.005749465
ENSG00000168874	ATOH8	0.848818574	0.005901578
ENSG00000174473	GALNTL6	-1.001236023	0.005902948
ENSG00000266074	BAHCC1	-0.64739927	0.005919479
ENSG00000204740	MALRD1	-1.200847317	0.005925588
ENSG00000092969	TGFB2	1.190774446	0.005937038
ENSG00000180185	FAHD1	-0.553978655	0.006076574
ENSG00000106635	BCL7B	0.658565814	0.006180558
ENSG00000183049	CAMK1D	0.740456408	0.006628651
ENSG00000076356	PLXNA2	1.060161503	0.007071145
ENSG00000116016	EPAS1	0.710660721	0.007310133
ENSG00000177707	NECTIN3	0.928397449	0.008001667
ENSG00000164236	ANKRD33B	1.298113022	0.008438208
ENSG00000159840	ZYX	0.59033282	0.008713077
ENSG00000196814	MVB12B	0.82873454	0.008713077
ENSG00000185442	FAM174B	0.654451371	0.009151961
ENSG00000152270	PDE3B	0.715891537	0.00947575
ENSG00000069667	RORA	1.162401449	0.009672809
ENSG00000105137	SYDE1	0.649403117	0.009693199
ENSG00000286523	-	-2.077053571	0.009696514
ENSG00000181104	F2R	1.173707777	0.009853825
ENSG00000103485	QPRT	-1.653053249	0.009875314
ENSG00000198948	MFAP3L	-1.016024851	0.009875314
ENSG00000069020	MAST4	0.736737671	0.010420293
ENSG00000185736	ADARB2	2.422037065	0.010472457
ENSG00000139793	MBNL2	0.666241554	0.010781585
ENSG00000082684	SEMA5B	1.027668116	0.010781585
ENSG00000224899	LINC02830	1.527838579	0.010781585
ENSG00000116774	OLFML3	-1.304608755	0.010805549
ENSG00000147955	SIGMAR1	-1.157881403	0.010805549
ENSG00000180938	ZNF572	-0.619719575	0.011983847
ENSG00000076716	GPC4	0.918676078	0.011983847
ENSG00000150907	FOXO1	0.752117822	0.012268421

ENSG00000164850	GPER1	-0.96672716	0.012499516
ENSG00000214293	APTR	0.66005604	0.012852333
ENSG00000157404	KIT	-1.328505763	0.012897939
ENSG00000141527	CARD14	-0.598080812	0.012995421
ENSG00000171617	ENC1	0.563968494	0.012995421
ENSG00000144290	SLC4A10	-1.159528989	0.013028973
ENSG00000145451	GLRA3	1.055015513	0.013028973
ENSG00000237187	NR2F1-AS1	3.764906575	0.014042161
ENSG00000090661	CERS4	-0.758894532	0.014576441
ENSG00000101986	ABCD1	-0.759319392	0.01484599
ENSG00000003096	KLHL13	1.071467939	0.01484599
ENSG00000197696	NMB	1.008653941	0.015601172
ENSG00000083720	OXCT1	-0.816172872	0.015972041
ENSG00000214193	SH3D21	-0.504225322	0.015972041
ENSG00000161642	ZNF385A	-0.473793931	0.015972041
ENSG00000020181	ADGRA2	1.462585829	0.015972041
ENSG00000153993	SEMA3D	2.385145542	0.015972041
ENSG00000159167	STC1	-0.742604055	0.01603399
ENSG00000128039	SRD5A3	0.777479384	0.016155137
ENSG00000082781	ITGB5	0.741839788	0.016451458
ENSG00000102699	PARP4	0.576630835	0.016743787
ENSG00000188959	C9orf152	-1.372582578	0.017195686
ENSG00000168679	SLC16A4	-1.372917814	0.017261245
ENSG00000151718	WWC2	0.738561796	0.017261245
ENSG00000136238	RAC1	0.575233958	0.017618152
ENSG00000091409	ITGA6	1.16162003	0.017817829
ENSG00000185924	RTN4RL1	-0.553046234	0.018107152
ENSG00000257671	KRT7-AS	1.165481696	0.01907405
ENSG00000134504	KCTD1	0.624744539	0.019107605
ENSG00000127955	GNAI1	-1.027798495	0.019242308
ENSG00000112183	RBM24	-0.990531229	0.019242308
ENSG00000095321	CRAT	-0.865522502	0.019242308
ENSG00000128274	A4GALT	-0.829521154	0.019828877
ENSG00000196636	SDHAF3	-1.397373821	0.020109986
ENSG00000172794	RAB37	-0.977071402	0.020109986
ENSG00000214078	CPNE1	-0.656533189	0.020109986
ENSG00000166833	NAV2	1.535642143	0.020119476
ENSG00000253276	CCDC71L	0.563147043	0.020319347
ENSG00000131746	TNS4	-1.171019047	0.020401967
ENSG00000198246	SLC29A3	0.598720205	0.020600083
ENSG00000172551	MUCL1	-0.924446266	0.022069823
ENSG00000139289	PHLDA1	0.748078573	0.022069823
ENSG00000162496	DHRS3	1.124026074	0.022069823
ENSG00000115290	GRB14	-0.591415986	0.022820118
ENSG00000230882	-	1.058086599	0.022820118

ENSG00000249700	SRD5A3-AS1	0.999653999	0.023737661
ENSG00000164932	CTHRC1	-1.184322986	0.025845308
ENSG00000106617	PRKAG2	0.63438887	0.026066954
ENSG00000117525	F3	1.150824104	0.026129253
ENSG00000106638	TBL2	0.540525634	0.026634815
ENSG00000127948	POR	0.628814201	0.028129504
ENSG00000136383	ALPK3	0.946763384	0.028129504
ENSG00000166974	MAPRE2	1.020963791	0.028129504
ENSG00000196428	TSC22D2	0.570377694	0.028296915
ENSG00000232533	-	0.871876528	0.028296915
ENSG00000144366	GULP1	0.872570518	0.02856424
ENSG00000149573	MPZL2	0.616046997	0.02874668
ENSG00000079805	DNM2	0.50576267	0.029296616
ENSG00000058091	CDK14	0.863235517	0.029529631
ENSG00000111058	ACSS3	0.877215967	0.029529631
ENSG00000153904	DDAH1	-0.750222768	0.029722896
ENSG00000105877	DNAH11	1.069564245	0.03040863
ENSG00000139352	ASCL1	-1.227245488	0.030814065
ENSG00000186187	ZNRF1	0.518411577	0.030894038
ENSG00000071859	FAM50A	-0.607014788	0.031228581
ENSG00000079257	LXN	0.619899203	0.031929235
ENSG00000123095	BHLHE41	0.828581841	0.033039427
ENSG00000145824	CXCL14	-4.897880799	0.033334657
ENSG00000117868	ESYT2	0.415393014	0.033334657
ENSG00000232931	LINC00342	1.023885812	0.033334657
ENSG00000143878	RHOB	-0.526929328	0.03444086
ENSG00000283538	-	1.063049627	0.034882785
ENSG00000258667	HIF1A-AS3	0.937183134	0.034945109
ENSG00000230221	-	3.954877082	0.034999533
ENSG00000154274	C4orf19	0.568342414	0.037211785
ENSG00000164266	SPINK1	2.19454636	0.037527932
ENSG00000185033	SEMA4B	0.573268786	0.037692183
ENSG00000136830	NIBAN2	0.610188212	0.037692183
ENSG00000085831	TTC39A	-0.494439943	0.039322054
ENSG00000241258	CRCP	0.406772042	0.039396988
ENSG00000163364	LINC01116	0.801399256	0.039396988
ENSG00000178821	TMEM52	0.803856407	0.039396988
ENSG00000127129	EDN2	0.879138707	0.039396988
ENSG00000189212	DPY19L2P1	1.729940482	0.039396988
ENSG00000085741	WNT11	1.758298528	0.039396988
ENSG00000168269	FOXI1	-1.251919043	0.03986553
ENSG00000121671	CRY2	-0.552356356	0.03986553
ENSG00000149929	HIRIP3	-0.526245111	0.03986553
ENSG00000105711	SCN1B	0.641812557	0.03986553
ENSG00000177283	FZD8	0.812610501	0.040268156

ENSG00000035664	DAPK2	0.407928743	0.041586398
ENSG00000132535	DLG4	-1.299819499	0.041646165
ENSG00000181588	MEX3D	0.670043208	0.041865833
ENSG00000146151	HMGCLL1	-1.144578957	0.042257282
ENSG00000100842	EFS	-1.633628557	0.043628975
ENSG00000179083	FAM133A	-1.014674402	0.043628975
ENSG00000142871	CCN1	0.561051523	0.043628975
ENSG00000146757	ZNF92	0.752250317	0.043628975
ENSG00000087053	MTMR2	0.901623924	0.043831175
ENSG00000151655	ITIH2	-1.536930979	0.044104959
ENSG00000156463	SH3RF2	0.767569119	0.044104959
ENSG00000008311	AASS	2.265648385	0.044348026
ENSG00000123836	PFKFB2	0.608542308	0.044884674
ENSG00000260401	-	0.768734559	0.045235838
ENSG00000181085	MAPK15	-0.725214109	0.04634039
ENSG00000086619	ERO1B	0.835003061	0.046831615
ENSG00000136828	RALGPS1	0.58962825	0.046972194
ENSG00000006576	PHTF2	0.584285468	0.047111228
ENSG00000203971	-	4.945148389	0.047615473
ENSG00000071575	TRIB2	0.812972534	0.047985894
ENSG00000185973	TMLHE	-0.534667382	0.048110254
ENSG00000157502	PWWP3B	-1.519630917	0.048310049
ENSG00000259527	LINC00052	-2.729850582	0.048942107
ENSG00000182985	CADM1	0.705305357	0.048942107
ENSG00000149591	TAGLN	0.832459222	0.048942107
ENSG00000115602	IL1RL1	1.750644752	0.049525138
ENSG00000186197	EDARADD	1.300889719	0.049677344

Supplementary table S3. Sequences of sgRNA used for CRISPR/Cas9 as well as primers used for real time PCR

**Gene**	**Primer Pair**
sgRNA1(Cas9wt)	F: 5’-**CACCg**GGACGAGATGAAGGCGTCTG-3’<br>R: 5’-**AAAC**CAGACGCCTTCATCTCGTCCc-3’
sgRNA2.1 (Cas9n)	F: 5’-**CACCg**TCATCTCGTCCTCTGACTTC-3’<br>R: 5’-**AAAC**GAAGTCAGAGGACGAGATGAc-3’
sgRNA2.2 (Cas9n)	F: 5’-**CACCg**TGCCACCGTGCTCACCGCCC-3’<br>R: 5’-**AAAC**GGGCGGTGAGCACGGTGGCAc-3’
genomic MB	F: 5'-TGGGAAGACAGGGAGCTAAA-3'<br>R: 5'-GCTCTGCCATTATCCACCTC-3'
TWIST	F: 5'-GGAGTCCGCAGTCTTACGAG-3'<br>R: 5'-TCTGGAGGACCTGGTAGAGG-3'
SNAIL	F: 5'-GACCACTATGCCGCGCTCTT-3'<br>R: 5'-TCGCTGTAGTTAGGCTTCCGATT-3'
SLUG	F: 5'-TTCGGACCCACACATTACCT-3'<br>R: 5'-GCAGTGAGGGCAAGAAAAAG-3'
FN1	F: 5'-TCGCCATCAGTAGAAGGTAGCA-3'<br>R: 5'-TACTTTCTTGATTTTCTTCCACAGCATA-3'

ZEB1	F: 5'-TACAGAACCCAACTTGAACGTCACA-3'<br>R: 5'-GATTACACCCAGACTGCGTCACA-3'
MMP3	F: 5'-CAACAAGAGCTAAGTAAAGCCAGTGG-3'<br>R: 5'-CTAGATATTTCTGAACAAGGTTCATGCT-3'
PI3K CA	F: 5'-AGTAGGCAACCGTGAAGAAAAG-3'<br>R: 5'-GAGGTGAATTGAGGTCCCTAAGA-3'
PI3K CB	F: 5'-CTGCCTGCGACAGATGAGTG-3'<br>R: 5'-TCCGATTACCAAGTGCTCTTTC-3'
FASN	F: 5'-CATCCAGATAGGCCTCATAGAC-3'<br>R: 5'-CTCCATGAAGTAGGAGTGGAAG-3'
EGLN1	F: 5'-CAAATGGAGATGGAAGATGTGTG-3'<br>R: 5'-AATGTCAGCAAACTGGGCTTT-3'
EPO	F: 5'-ATGTGGATAAAGCCGTCAGT-3'<br>R: 5'-AGTGATTGTTCGGAGTGGAG-3'
CITED2	F: 5'-ACCATCACCCTGCCCACC-3'<br>R: 5'-CGTAGTGTATGTGCTCGCCCA-3'
SOD1	F: 5'-TACAAAGACAGGAAACGCTGG-3'<br>R: 5'-CCTCAGACTACATCCAAGGGAA-3'
SOD2	F: 5'-AGGTGACTCTAACTTCCCTGGC-3'<br>R: 5'-CCCACAAGCACAGAAATAAAGGAGA-3'
SOD3	F: 5'-CGTTCCTGGGCTGGCTGGGT-3'<br>R: 5'-ATGGCTGGAGTCGGGCACCTTT-3'
GPX3	F: 5'-AGGTATGCGTGATTGTGTGTGT-3'<br>R: 5'-GGAGAACTGGAGAGAAAGGGTTG-3'
GPX4	F: 5'-CGCTGTGGAAGTGGATGAAGA-3'<br>R: 5'-CTTGTCGATGAGGAACTTGGTGAA-3'
CAT	F: 5'-GACATTACCAAATACTCCAAGGCAA-3'<br>R: 5'-AACCCGATTCTCCAGCAACA-3'
ACTB	F: 5'-CTGGAACGGTGAAGGTGACA-3'<br>R: 5'-AAGGGACTTCCTGTAACAACGA-3'
